# Systems Biology in ELIXIR: modelling in the spotlight

**DOI:** 10.12688/f1000research.126734.1

**Published:** 2022-11-07

**Authors:** Vitor Martins dos Santos, Mihail Anton, Barbara Szomolay, Marek Ostaszewski, Ilja Arts, Rui Benfeitas, Victoria Dominguez Del Angel, Polonca Ferk, Dirk Fey, Carole Goble, Martin Golebiewski, Kristina Gruden, Katharina F. Heil, Henning Hermjakob, Pascal Kahlem, Maria I. Klapa, Jasper Koehorst, Alexey Kolodkin, Martina Kutmon, Brane Leskošek, Sébastien Moretti, Wolfgang Müller, Marco Pagni, Tadeja Rezen, Miguel Rocha, Damjana Rozman, David Šafránek, Rahuman S. Malik Sheriff, Maria Suarez Diez, Kristel Van Steen, Hans V Westerhoff, Ulrike Wittig, Katherine Wolstencroft, Anze Zupanic, Chris T. Evelo, John M. Hancock

**Affiliations:** 1 Laboratory of Bioprocess Engineering, Wageningen University & Research, Wageningen, 6708 PB, The Netherlands; 2 Department of Biology and Biological Engineering, National Bioinformatics Infrastructure Sweden, Science for Life Laboratory, Chalmers University of Technology, Gothenburg, SE-41258, Sweden; 3 Division of Infection and Immunity, School of Medicine, Cardiff University, Cardiff, UK; 4 Luxembourg Centre for Systems Biomedicine (LCSB), University of Luxembourg, Belvaux, L-4367, Luxembourg; 5 Maastricht Centre for Systems Biology (MaCSBio), Maastricht University, Maastricht, 6200 MD, The Netherlands; 6 National Bioinformatics Infrastructure Sweden (NBIS), Science for Life Laboratory, Department of Biochemistry and Biophysics, Stockholm University, Stockholm, Sweden; 7 Research and Platforms Department, Genopole, Evry-Courcouronnes, 91030, France; 8 Faculty of Medicine, Institute for Biostatistics and Medical Informatics, Centre ELIXIR-SI, University of Ljubljana, Ljubljana, SI-1000, Slovenia; 9 Systems Biology Ireland, School of Medicine, University College Dublin, Dublin, 4, Ireland; 10 Department of Computer Science, The University of Manchester, Manchester, M13 9PL, UK; 11 Heidelberg Institute for Theoretical Studies - HITS, Heidelberg, 69118, Germany; 12 Department of Biotechnology and Systems Biology, National Institute of Biology, Ljubljana, SI-1000, Slovenia; 13 ELIXIR Hub, Hinxton, Cambridge, CB10 1SD, UK; 14 European Molecular Biology Laboratory, European Bioinformatics Institute (EMBL-EBI), Hinxton, Cambridge, CB10 1SD, UK; 15 Scientific Network Management SL, Barcelona, 08015, Spain; 16 Metabolic Engineering & Systems Biology Laboratory, Institute of Chemical Engineering Sciences, Foundation for Research & Technology - Hellas (FORTH/ICE-HT), Patras, 26504, Greece; 17 Laboratory of Systems and Synthetic Biology, Wageningen University & Research, Wageningen, 6708WE, The Netherlands; 18 ISBE.NL, VU University of Amsterdam, Amsterdam, The Netherlands; 19 Competence Center for Methodology and Statistics; Transversal Translational Medicine, Translational Medicine Operations Hub, Luxembourg Institute of Health, Strassen, L-1445, Luxembourg; 20 Department of Bioinformatics - BiGCaT, NUTRIM, Maastricht University, Maastricht, 6200 MD, The Netherlands; 21 SIB Swiss Institute of Bioinformatics, Lausanne, Switzerland; 22 Faculty of Medicine, University of Ljubljana, Ljubljana, SI-1000, Slovenia; 23 Centre of Biological Engineering, University of Minho, Braga, Portugal; 24 Faculty of Informatics, Masaryk University, Brno, 602 00, Czech Republic; 25 BIO3 - Systems Genetics, GIGA-R Medical Genomics, University of Liege, Liege, 4000, Belgium; 26 BIO3 - Laboratory for Systems Medicine, Department of Human Genetics, KU Leuven, Leuven, 3000, Belgium; 27 Leiden Institute of Advanced Computer Science, Leiden University, Leiden, 2333 CA, The Netherlands

**Keywords:** Systems Biology, Systems Medicine, ELIXIR Communities, Biomolecular Models, Network Biology, FAIR, Biological data, Biotechnology

## Abstract

In this white paper, we describe the founding of a new ELIXIR Community - the Systems Biology Community - and its proposed future contributions to both ELIXIR and the broader community of systems biologists in Europe and worldwide. The Community believes that the infrastructure aspects of systems biology - databases, (modelling) tools and standards development, as well as training and access to cloud infrastructure - are not only appropriate components of the ELIXIR infrastructure, but will prove key components of ELIXIR’s future support of advanced biological applications and personalised medicine.

By way of a series of meetings, the Community identified seven key areas for its future activities, reflecting both future needs and previous and current activities within ELIXIR Platforms and Communities. These are: overcoming barriers to the wider uptake of systems biology; linking new and existing data to systems biology models; interoperability of systems biology resources; further development and embedding of systems medicine; provisioning of modelling as a service; building and coordinating capacity building and training resources; and supporting industrial embedding of systems biology.

A set of objectives for the Community has been identified under four main headline areas: Standardisation and Interoperability, Technology, Capacity Building and Training, and Industrial Embedding. These are grouped into short-term (3-year), mid-term (6-year) and long-term (10-year) objectives.

## Executive summary

This white paper presents the future strategy of the new ELIXIR Systems Biology Community. This emerging ELIXIR Community was established upon the recommendation of ELIXIR’s Systems Biology Focus Group to develop and coordinate ELIXIR’s interactions with the broader systems biology community. **The infrastructure aspects of systems biology - databases, tools and standards development, as well as training and access to cloud infrastructure - are not only appropriate components of the ELIXIR infrastructure, but will prove effective drivers of ELIXIR’s future support of advanced biological applications and personalised medicine.**

Systems biology is defined here as: modelling and understanding living systems in terms of their thousands of molecular interaction properties using a wide range of approaches, which can be further classified as bottom-up (starting from the molecular components) or top-down (starting from system behaviour). A key feature of systems biology is that, because of the complexity of the systems that are studied and the variety of data that is collected, it often requires collaboration between different laboratories and between computational biologists and experimentalists, often asynchronous in both space and time (e.g. through the literature and databases). It is essential that all the components that go towards building a systems model, from datasets and data collection methods to the models themselves, are FAIR (Findable, Accessible, Interoperable and Reusable) ( Wilkinson *et al.*, 2016).

Historically, in Europe and worldwide, there have been significant investments in the development and applications of systems biology (see Table 1 & Table 2). In Europe, this culminated in the establishment of the ESFRI ISBE (Infrastructure Systems Biology Europe); however, this initiative fell short of obtaining sufficient support from member states. ELIXIR’s Systems Biology Community will build upon some of the strands of the work in ISBE, as well as work that has been taking place within ELIXIR’s Communities and Platforms.

**Table 1.** Summary of European initiatives in Systems Biology.


**H2020 projects**	Projects EmpowerPutida, P4SB, Shikifactory100, DD-DECAF, SINFONIA, BIOS and SafeChassis (all industrial biotechnology), BioRoboost (standards SynBio) were / are all efforts connected to Metabolic Engineering / Industrial Biotech; PoLiMeR (Polymers in the Liver: Metabolism and Regulation, ITN); MESI-STRAT (Systems Medicine of Metabolic-Signaling Networks - A New Concept for Breast Cancer Patient Stratification); EPIPredict- (Systems Biology around the epigenetics of Estrogen Receptor-mediated breast cancer). REPO-TRIAL (Systems Medicine and drug repurposing) ADAPT (Accelerated Development of multiple-stress tolerAnt PoTato) EERA CoBioTech cofund action for s *ystems biology* and *synthetic biology* for *industrial biotechnology;* previous ERANets include ERASysAPP and SysMO. ERA-Net Cofund Scheme ERACoSysMed aims to enhance the implementation of Systems Biology approaches in medical concepts, research, and practice throughout by structuring, coordinating, and integrating national efforts and investments. CORBEL ( https://www.corbel-project.eu/) - Coordinated Research Infrastructures Building Enduring Life-science Services, where ISBE provided modelling services coordinated with other Research Infrastructures.
**ESFRI & other** ** (national) ** **research** ** infrastructure**	ELIXIR Microbial Biotechnology Community, on the specific ESFRI project www.IBISBA.eu (industrial biotechnology, many aspects related to workflow, data and models intertwined very tightly) ISBE (Infrastructure Systems Biology Europe, see below) UNLOCK (An open infrastructure for exploring new horizons for research on microbial communities), a Dutch infrastructure on the national roadmap. A worldwide network of biofoundries of special relevance to synthetic biology and industrial biotechnology, https://biofoundries.org/
**IMI H2020** ** projects**	Drug Disease Model Resources ( DDMoRe) Enhancing Translational Safety Assessment through Integrative Knowledge Management ( eTRANSAFE) Translational quantitative systems toxicology to improve the understanding of the safety of medicines ( transQST)
**EASYM**	EASyM is a charitable association open to everyone with an interest in personalised medicine and Systems Medicine.
**VPH**	The virtual physiological human ( VPH) is a long-standing and successful activity focusing on modelling physiology, such as that of the heart and of diabetes. It is much less molecular than mainstream Systems Biology (but of course not less relevant thereby).
**EU-STANDS4PM**	European standardization framework for data integration and data-driven *in silico* models for personalised medicine.
**Disease Maps**	An open community effort to comprehensively represent disease mechanisms for various diseases.
**ITFoM**	For a long time now initiatives aiming to make comprehensive, molecules-up, models of entire organisms, including the human, have been brewing. These even made it into a candidate flagship programme of the EC but did not receive funding. Yet the European Commission is still drafting a roadmap for such a program, called EC initiative ‘ Human Digital Twin’, the European Commission is currently drafting a roadmap
**BioModels**	BioModels is an EMBL-EBI based repository for curated models, with a focus on SBML-based models, but also providing models in other representations.
**Metabolic Atlas**	Metabolic Atlas is a freely available repository and tool for visualisation and exploration of open-source genome-scale metabolic models (GEMs), particularly for human and model organisms, developed by the Nielsen Lab at Chalmers University of Technology. The web portal is developed open-source, it integrates GEMs benefiting from community-driven curation towards FAIR models, and it further presents a number of tissue-, cell line- and disease-specific GEMs for usage in systems medicine approaches.
**MetaNetX/** **MNXref**	MetaNetX is a unified namespace of metabolites and biochemical reactions developed to bring together models and resources published by other groups. It is distributed under open-source licence as a database (MNXref). In addition, a standalone software suite (MNXtools) will soon be released to critically assess and suggest improvement for Genome-Scale Metabolic Models, w.r.t to biochemistry.

**Table 2.** Summary of international initiatives and resources in Systems Biology.


**COMBINE**	COmputational Modelling in BIology NEtwork ( COMBINE) that coordinates the development of modelling standards, e.g. SBML, CellML, SED-ML, SBGN, SBOL, and others.
**COVID-19 Disease Maps**	COVID-19 Disease Map, aims to establish a knowledge repository of molecular mechanisms of COVID-19 as a broad community-driven effort with contributions from the Disease Maps community, WikiPathways, and Reactome.
**LiSyM (Germany)** **LiSyM-Cancer**	Liver Systems Medicine Network ( LiSyM): Striving to develop non-invasive methods for diagnosing and treating NAFLD by combining mathematical modelling and biological research. Follow-up initiative LiSyM- Cancer started in July 2021.
**Center for reproducible** ** biomedical modelling**	Reproducible Biomedical modelling aims to enable larger and more accurate systems biology models, as well as their applications to science, bioengineering, and medicine, by enhancing their understandability, reusability, and reproducibility.
**Interagency Modelling** ** and Analysis Group** ** (IMAG) - Multiscale ** **Modelling Consortium**	IMAG is a government group of program officials from multiple federal government agencies supporting research funding for modelling and analysis of biomedical, biological, and behavioural systems. The IMAG wiki supports the activities of the Multiscale Modelling (MSM) Consortium and other IMAG agency- supported research consortia that focus on modelling and analysis projects. MSM consortium is focused on multiscale modelling of biomedical, biological, and behavioural systems. An example of an MSM subgroup is the recently formed Multiscale modelling and Viral Pandemics to tackle ongoing and future viral pandemics.
**INCOME**	INtegrative COllaborative modelling in systems MEdicine
**BioSys ANR call in** ** France**	Funding of several excellence initiatives in France, including Institutes of convergence and Laboratories of Excellence closely related to Systems Biology.
**ERAnet Sysbio call**	To promote multidimensional and complementary European Systems Biology projects, programmes, and research initiatives on a number of selected research topics on applied translational Systems Biology.
**JWS Online**	A transnational live model repository initiated in Stellenbosch (South Africa), and then extended to Amsterdam and Manchester. JWS Online has been integrated into FAIRDOM and has been funded by various South African, Dutch, German, and UK grants.
**COPASI**	A software tailor-made for (stochastic) rate-balance-equation modelling and analysis of biochemical reaction and signalling networks. COPASI has been and is funded by various grants mostly from the UK, German, and US governments, and is an ELIXIR service, fully integrated with Systems Biology standardisation.
**FAIRDOM**	A community ( FAIRDOM), software platform ( FAIRDOM-SEEK) ( Wolstencroft *et al.*, 2015), and public resource ( FAIRDOMHub) ( Wolstencroft *et al.*, 2017) serving the asset management needs of Systems Biology projects are created and hosted by several ELIXIR Nodes. Of the 140+ instances of the FAIRDOM-SEEK many serve Sys Bio (e.g. Leipzig Health Atlas, LiSyM, IBISBAHub). The FAIRDOM-SEEK is the platform used by the Industrial Biotech ESFRi IBISBA (IBISBAHub).
**WikiPathways**	WikiPathways is an open database of biological pathways maintained by and for the scientific community ( Martens *et al.*, 2021). WikiPathways is managed by the Gladstone Institute in San Francisco and Maastricht University in the Netherlands. The project is supported by grants in the US and NL, and it is also an ELIXIR service.
**COLOMOTO**	COLOMOTO is a consortium of research groups interested in logical modelling: modellers, curators and developers of methods and tools. The consortium works on the definition of standards for model representation and interchange (especially the SBML qual format), and on the comparison of methods, models and tools.

The Community has identified seven key challenges for systems biology in the short to medium term that can be addressed in part by ELIXIR, each with their own identified sub-challenges:

1. **Barriers to the wider uptake of systems biology** (the challenge of providing well-parameterized systems biology models across the breadth of the life sciences; lack of data that can be used to accurately define the model parameter values; lack of standardisation and interoperability of systems biology; the resistance to mathematical modelling that is still present in biological and medical sciences)
2. **Linking new and existing data to systems biology models** (FAIR generation of data well-suited to use in modelling and linking data and models in a FAIR way)
3. **Interoperability of systems biology resources** (descriptions and annotations of data, models and their content need to follow coherent terminologies and ontologies; models, workflows, and data require FAIR, state-of-the-art computational infrastructure and tools for storage, access, and efficient use; interlinking standard models with models expressed in scientific programming and general purpose languages; interlinking descriptive and predictive models; trained experts to develop and curate resources that are user friendly and accessible; availability and accessibility of existing computational analysis methods and their interoperability with relevant modelling approaches)
4. **Further development and embedding of systems medicine** (identification and modelling of network structures that are prognostic and predictive; integration with related systems such as microbes or expososomes; personalising models using patient data; clinically validating models; addressing ELSA (Ethical, Legal and Social Aspects) for clinical, sensitive data; interfacing with epidemiology)
5. **Provisioning of modelling as a service** (provisioning of sophisticated data resources, tools and rich standards that are useful both for data mining and experimental design; availability of a pool of experts; availability of blueprint models to help with building new models)
6. **Capacity building and training** (different trainee backgrounds; need for a systems biology learning path and broad promotion of the integrated systems biology framework; availability and use of standardised datasets in training materials; need for a broad training expertise)
7. **Need to support industrial and societal embedding** (addressing, among others, pharmacology; toxicology; diagnostics; synthetic biology; agronomy; including microbial biotechnology and the bio-economy)

Many areas of relevance to systems biology are already embedded into activities of ELIXIR’s Platforms, Communities and Focus Groups (see the Systems Biology within ELIXIR section below) and have the potential for further developments. In particular we identify the following:

**Data platform:** ELIXIR Core Data Resources and Deposition Databases are already highly used by the systems biology community. For example, data from BRENDA ( Chang *et al.*, 2021), STRING ( Szklarczyk *et al.*, 2021), and Reactome ( Gillespie *et al.*, 2022) are essential both for construction of molecular pathways and parameterization of molecular reactions. There is potential for the Systems Biology Community to add new data resources to the ELIXIR list of services. Engagement of the Platform with curation efforts like BioModels ( Malik-Sheriff *et al.*, 2020) can improve the FAIRness of systems biology-related data resources.

**Tools platform:** bio.tools ( Ison *et al.*, 2016) and the workflow hub ( Goble *et al.*, 2021) are invaluable resources for finding computational tools for systems biology. The ELIXIR Systems Biology Community aims to ensure relevant tools and workflows are added to these resources.

**Compute platform:** This Platform has strong links to the European Open Science Cloud which will be important for future instantiation and simulation of extensive, multiscale models in the cloud.

**Interoperability platform:** The Interoperability Platform’s Recommended Interoperability Resources help to improve the FAIRness of systems biology-related data, tools and models, while the Platform’s standards mapping resources such as BridgeDb ( van Iersel *et al.*, 2010), identifiers.org ( Wimalaratne *et al.*, 2018) and OLS ( Jupp *et al.*, 2015) facilitate better interoperability and integration of data and models. The systems biology community at large has also developed its own standards, like SBGN (Systems Biology Graphical Notation) and SBML (Systems Biology Markup Language), which we want to better connect with the abovementioned resources. The FAIRDOM platform ( Wolstencroft *et al.*, 2017) (part of the ELIXIR CONVERGE data management toolkit) provides a collaborative community space for the FAIR integration of data and models in their experimental context.

**Training platform:** Better and more extensive training in systems biology tools and methods, e.g. including the development of the new training materials, providing a repository for training materials and an annotation process for training materials, will be essential for the wide uptake of systems biology methodologies.

**Communities:** Many Communities already cover systems biology aspects and may well benefit from and provide data, tools and expertise for the modelling of various aspects in their systems. From a technological perspective, the *Galaxy Community* will play an important role in the integration of omics data and systems biology tools into workflows. Related to this, the *Metabolomics Community* has already begun work on standardising fluxomics workflows. The *Microbial Biotechnology Community* has a strong interest in systems biology as it aims to contribute to addressing standardisation and other issues in relation to models and their applicability. Other Communities, such as *Plant Sciences*, *Microbiome* (a new Community emerging from the Marine Metagenomics Community), *Food and Nutrition* and *Toxicology*, have potential to develop and deploy systems biology applications as part of their work towards understanding of their systems under study. It is foreseen that the Human Data Communities, especially the *Federated Human Data* and *Rare Diseases* Communities, will provide data, tools and expertise for the modelling of human disease.

**Focus groups:** The *Machine Learning*, *EOSC (European Open Science Cloud)* and *Registries* Focus Groups will be instrumental in the advancement of new techniques for model development and the implementation of systems models in a cloud environment (making use of registries to make data, tools and workflows FAIR). FAIRness will also be improved by working with the *Biocuration* and *FAIR Training* Focus Groups. Large-scale systems biology modelling of interactions and evolution of populations and ecosystems is likely to increase, and it is expected that the *Biodiversity* Focus Group will mediate the interactions of the Community with such efforts.

The Community has developed a plan for future aims and objectives on short (3 years), mid (6 years) and long-term (10 years) timescales, revolving around four pillars: Standardisation & Interoperability, Technology, Training & Capacity building, and Embedding.

**Short term:**

- Better support of existing standards in model repositories;
- Build upon systems biology models to improve the design of experiments that lead to the generation of higher quality, quantitative, FAIR data;
- Address specific challenges for human modelling, which include: working with compartments; model validation through standardised phenotypes; initial interfaces for multi-level modelling and integration across scales; multi-tissue evaluations; extrapolations from single cell analysis to tissue level; microbiome - host interactions; integrating sensitive personal data into models for personalised medicine;
- Establish approaches for model exchange, building on existing resource developments in the FAIR data landscape (e.g. FAIR data points; JWS-Online), BioModels and ModeleXchange;
- Understanding how big/smart data meets models meaningfully;
- Intertwining temporal and spatial modelling appropriately;
- Improved interoperability of modelling, simulation and analysis tools;
- Interfacing to synthetic biology through model-based design and model-based-learning strategies;
- Pre-screen trainees prior to training events to make recommendations for courses to be followed in the context of the event;
- Integrate new systems biology courses into TeSS and co-promote them with existing TeSS courses;
- Strengthen synergies with the other ELIXIR Communities, e.g. via joint training events;
- Together with the training platform set up a "gap analysis survey" to find out the strengths and needs for each ELIXIR Node;
- Implement turnkey solutions for different Nodes (specific training, capacity building, staff exchanges, knowledge exchanges and so on);
- Establish Key Performance Indicators (KPIs) to measure the impact of different actions.

**Mid-term:**

- Improved standardisation of meta-data to describe time-series, functional and imaging data such as to facilitate their integration in computational models; Providing a link between existing models, datasets, and analysis tools for easy access to relevant data;
- Good strategies (including training) to improve reproducibility, credibility, and validation of models and to assess the efficiency of tools, leading to the development of quality marks, thereby increasing the quality of workflow outcomes;
- Developing the basis for theoretical and practical multi-scale modelling frameworks;
- Providing the basis for developing Digital Twins (microbes, bioreactors, organs, organisms, ecosystems);
- Extend the use of synthetic and standardised datasets in most systems biology training events;
- Support current and future trainers via Train the Trainer ELIXIR events;
- Continue and review the process of gap analysis survey and KPIs to include SME and industry;
- Involve small and medium-size enterprises in the capacity building process;
- Identify new areas, themes and challenges in systems biology.

**Long-term:**

- Improved interoperability of data and models to enable FAIR model connection and integration (at different scales) so as to facilitate the development of multi-scale modelling frameworks;
- Development of Digital Twin methodologies that provide sufficiently accurate, real-time and dynamic depictions of physical biosystems (microbes, bioreactors, organs, organisms, ecosystems);
- Steer and modify processes, stratify patients and thereby support medical decision-making;
- Create a centralised repository of systems biology training materials aggregated by TeSS;
- Systematically review trends in systems biology and update training resources accordingly;
- Automation of the capacity building process for new partners (communities, countries, etc.).
- Increase uptake of systems biology methodologies by the communities of biologists, bioengineers and physicians;
- Increase the uptake of standards (e.g. for model and data reporting) by the world wide systems biology communities.

## Introduction

Systems biology aims to understand how biological functions **emerge** from interactions between the multiple components of living systems by modelling the (dynamics of) interactions and processes. It studies what makes **the** **whole different from the sum of its parts**.

Since modelling and understanding of living systems in terms of their thousands of molecular properties cannot be achieved in one step, systems biology comprises a variety of diverse, complementary approaches. **Bottom-up** systems biology starts from a limited number of components and studies the nonlinear mechanisms through which new properties emerge that are important for function. **Top-down** systems biology searches in multiple functional dimensions of the whole living system for correlations and patterns that clarify where functions have arisen from coherent behaviour of components. Top-down and bottom-up models should ultimately converge and predict all experimentally measured behaviour. **Multiscale** systems biology links systems biology at a smaller or faster scale (e.g. the cell) to systems biology at a greater size or longer time (the organism or the population). A key feature of systems biology is often the integration of data, often in large volumes of data that may be heterogeneous, at multiple scales, both in space and in time.

Due to their internal and external connectivity and nonlinearities, the study objects of systems biology are generally too complex to be handled by a single laboratory. They require a ‘worldwide laboratory without walls’, akin to the open science community. For instance, data and models that come from different expert groups dealing with a cell’s nucleus need to be integrated with data and models of that cell’s cytosol, as well as with data and models of neighbouring cells or tissues. The **data** **integration** inherent in systems biology requires metadata **standards** and ontologies, for computer-aided data exchange and integration, for computational software, for quality control, for experimental methods and for the reporting of experimental data. **Models** are required for experimental design and experiments are needed to validate them, whilst standards are required for their communication ( Nickerson *et al.*, 2016; Stanford *et al.*, 2015; Stanford *et al.*, 2019; Waltemath *et al.*, 2016). **High Performance** **Computing** (HPC) hardware with dedicated **software** is required to run complex models. In addition, a **capacity building** strategy is key in order to train dedicated experts in the field.

Model-driven **integration of heterogeneous and distributed data and knowledge** is instrumental to the reconstruction of emergent behaviour in systems biology. For this integration to be scientifically tractable or even possible, the data, as well as the processes of integration and the models themselves, must be **FAIR** to begin with. For the integration of data and knowledge in models, continual improvements in interoperability are needed to improve the link between the entities in the model and the FAIR data, e.g. from a gene expressed to an active enzyme with supporting literature evidence.

**Figure 1.**
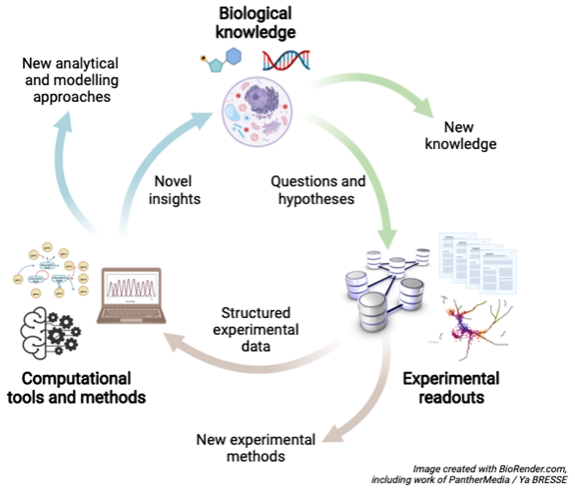
The systems biology “cycle”. To address a particular biological question, the cycle may start by data- and/or hypothesis-driven modelling of the system being addressed, followed by the generation of testable hypotheses, sets of predictions and design of the subsequent laboratory experiment. The experiment is carried out, data are generated and this is followed by computational analysis of these data, comparison of prediction to experiment and refinement of the model, which ultimately leads to gaining insights and generating new hypotheses. This cycle builds upon essential disciplines from Biology (the scientific question), Technology (experimentation and generation of data) and Computation (data analysis, modelling and prediction). Although the diagram is a cycle, it represents a spiral: with every turn data, knowledge and models increase, which could be seens as the cycle spiralling out of the plane of the paper/screen.

Many functions are critical to an organism’s evolutionary fitness and competitiveness. Because all components of any living organism are, often indirectly, connected, systems biology requires the **experimental collection, and then integration,** of results obtained from high-throughput genome-wide, or other holistic, methodologies (see Figure 1). Indeed, the **accelerating increase of omics data generation** has been a major driver of modern systems biology. In order to understand the complexity of living systems in realistic terms, much of systems biology engages in experimental analyses co-designed and analysed by mathematical modelling in a physical-chemical context.

The dependence on data from omics experiments, biochemistry and physiology mirrors the ‘ecosystem’ of data resources, tools, and Communities represented in ELIXIR. Data resources provide standardised and FAIR data of a particular kind. These data can be analysed through high-quality and well-characterised tools, often encapsulated in workflows. ELIXIR’s Communities then specialise in the standardisation and analysis of particular types of data, in their application to particular types of biological problems, or in underpinning technologies.

### Historical context: large-scale investment in systems biology in Europe and worldwide

In the early 2000s, the challenges and benefits of integrating experimental molecular biology with mathematics and informatics had already been demonstrated in systems biology. Reflecting its perceived impact on both life science research and economic development, systems biology research was supported by numerous European and global efforts. Accordingly, an infrastructure for systems biology ISBE (Infrastructure for Systems Biology in Europe) was added to the ESFRI roadmap in 2010. In subsequent years, and in the scope of an ESFRI preparatory phase, a science case and business plan were formulated with the goal of ISBE becoming a legal entity upon gathering support from the various member states involved. The efforts to establish ISBE as a stand-alone entity received the support of too few member states however, and ISBE was removed from the ESFRI roadmap in December 2021.

Whereas **ISBE** focused on **models for Life**, ELIXIR focuses on **data for Life**, but there is a continuum between the two: models rely on experimental data, and are often very tightly integrated with their supporting data. The same ambivalence applies to specific resources. An example is FAIRDOM, which was developed within ISBE as a follow-up from initial efforts within the ERA-Net Systems Biology, among others. Other examples are the BioModels repository, an ELIXIR Deposition Database, which stores models for Life, JWS Online, and perhaps the most visible part of ISBE, its Make me My Model component, mostly at ISBE.NL (see the ISBE deliverables ^1^).

### Development of the ELIXIR Systems Biology Community

In response to the issues faced by ISBE and its expected loss from the research infrastructure landscape in Europe, and the awareness that many aspects of systems biology were already features of ELIXIR resources, a Focus Group on Systems Biology was established in ELIXIR in April 2020, which held its first meeting in June of that year. The aim of this Focus Group was to recommend how ELIXIR should respond to the situation around ISBE and support systems biology. The Focus Group presented its report to the ELIXIR Heads of Nodes in May and September 2021 with the recommendation that a new Community in Systems Biology should be established within ELIXIR. This suggestion was supported by the ELIXIR Heads of Nodes and this white paper represents the agreed priorities and aims of this new grouping.

## Developing an ELIXIR Systems Biology Roadmap

### Key barriers for wider adoption of systems biology

The overarching long-term goal of the ELIXIR Systems Biology Community is to make systems biology modelling a central pillar of research in biology. In this vision, systems biological models are developed based on the understanding of the biological problem, are used to design biological experiments, and help with the interpretation of collected data. The combined results then allow the development of actionable solutions to the original biological problem.

While systems biology is present in all biological disciplines, it has so far not reached its full potential. To achieve this, systems biology needs to become more accessible to enable a broader community to benefit from its approaches. The ELIXIR Systems Biology Community has identified the main barriers that prevent wider adoption of systems biology tools and it aims to gradually eliminate them through its future activities.

One barrier is the lack of availability of well-parameterised systems biology models that are of immediate value for researchers in the wider community. Biologists study a great variety of organisms, each with a great variety of pathways, and physicians study a great variety of diseases in a great variety of tissues. While biological pathway/network resources and genome-scale metabolic maps have grown over the last decade and their usability for flux balance analyses has greatly improved, other approaches additionally require the setting of the much larger number of parameters inherent in rate equations. The values of these parameters differ between species, individuals and tissues. Consequently, the probability that any of the available kinetic, stochastic or multiscale models fits the experimental object a particular researcher is interested in is extremely small. Kinetic, stochastic, and multiscale models continue to be used only by modelling experts. There are many additional facets to this problem. The first is that systems biology models are rather difficult to build - both the understanding of the underlying biology and of skillful modelling are rarely present in the same person. While we can partly solve these issues via better training, the former is also due to a limitation that can only be diminished in incremental steps by increasing our understanding of biology. The second facet is the lack of data that can be used to accurately define a particular use case. As there are (almost) no two particular cases that have the same parameter values, this results in model predictions with wide confidence intervals and low predictive value. A resource such as BioModels Parameters ( Glont *et al.*, 2020) provides quick access to parameter values and ranges but is limited to models existing in the BioModels repository.

Another barrier lies in the insufficient standardisation and interoperability of systems biology, which often makes sharing and reuse a time-consuming challenge. The last decade has seen the creation and increased uptake of new systems biology standards, and increased sharing of standardised models with cross-referenced annotations in repositories, such as MetaNetX ( Moretti *et al.*, 2021) and BioModels. However, these do not yet sufficiently cover the areas of model parameter sharing and linking with experimental data. There has also been a huge increase in the development of systems biology software and tools that make use of the standards, but switching between tools remains cumbersome. Better interoperability would enable the building of efficient systems biology modelling pipelines. Here, the ELIXIR Systems Biology Community can learn from the recent advances in the standardisation of sharing of biological data and data analysis pipeline development, where the wider ELIXIR community has played an important role. 

Finally, we need to overcome the conceptual barrier to mathematical modelling that is still present in biomedical sciences. While biology is a mature science, in the sense that we have a decent understanding of the major processes that make it work, we mostly do not understand the details to make it truly applicable. To get at those details, and to take advantage of all the big and small datasets collected in the last decades, we need to build more and better systems biology models for better data interpretation, such that systems biology consistently demonstrates utility and becomes a part of all new biomedical studies. This needs to be done in ways that make the resulting models maximally accessible and usable by biomedical scientists.

### Linking new and existing data to mathematical models

While discussions of systems biology are often focused on the modelling part, there is very little that can be achieved without making use of high-quality data. Data is a source of knowledge necessary to develop the systems biology models, to parametrize them and to evaluate how well they describe the biological question at hand. However, beyond the data produced in their own group or by close collaborators, a modeller rarely finds data perfectly suited for the task at hand. Mostly, this is because the data needed simply do not exist, which is not surprising given the small section of biology we have analyzed experimentally so far. Often the data that would inform modelling are there, but extremely difficult to find or are not annotated well enough for subsequent integration with models. Data for parametrization of dynamic systems biology models are particularly problematic, as these need to be time courses that cover the entire dynamics of the modelled process: biological networks adapt over time.

There are therefore two types of challenges that need to be tackled when discussing data in systems biology. The first is a cultural challenge on the data generating side. If data are to be useful for systems biology modelling, then this needs to be taken into account already when the experiments are designed, or better yet, when the grant applications are written. The more systems biology modelling is included in the early stages of the project, the more we can count on a one-on-one connection between model and data.

The second challenge, and one that the ELIXIR is very well positioned to help meet, is linking the data to the models in a FAIR way. Ideally, in the future, this would mean that a model developer would be able to search and find data they need and an experimental scientist would be able to easily find that an existing model can be used to analyse the data obtained. In the last decade, a giant leap forward has been achieved by collecting increasingly large amounts of experimental data in FAIR repositories; however, the data has rarely been linked to modelling repositories. One potential way forward should be to make the data and modelling repositories mutually searchable. Another is to create data repositories that are aimed specifically at storing data that are useful for systems biology modelling. The first attempt in this direction is the recently established *datanator* repository ( Roth *et al.*, 2021). A second is FAIR joint model/data repositories, such as FAIRDOMHub ( Wolstencroft *et al.*, 2017) (which has strong links to both ISBE and ELIXIR), which allows interlinking of experimental data, computational models and simulation results in a project-centred approach.

### Interoperability of systems biology resources

The essential components of systems biology integrate well with the ELIXIR infrastructure. Although systems biology models have not been, until now, at the heart of the ELIXIR infrastructure, they share many essential properties with the components of ELIXIR. ELIXIR already makes workflows, in essence programs and tools, part of their portfolio. Models, workflows, and data require state-of-the-art computational infrastructure and tools for storage, access, and efficient use. All of this needs to be FAIR. They require highly educated and trained experts to develop and curate resources that are user-friendly and accessible. Additionally, systems biology models depend greatly on the quality and quantity of data for their construction and simulation.

**Data and metadata**

Metadata describing data and models with their items and entities need “minimal information” checklists for attributes to be listed. Descriptions and annotations of data, models and their content need to follow coherent terminologies and ontologies. This is a prerequisite for their integration into systems biology models. Models need standardised formatting and description (for comparison, for modularization, for integration/interlinkage into complex multiscale models, etc.). Standardised visualisation of models helps to share visual information (e.g. pathway diagrams, activity flows, entity relations) on the models in a consistent way.

There is no single tool that can encompass all aspects of systems biology. Being an interdisciplinary endeavour, systems biology projects span multiple specialities, multiple scales, multiple experimental methods, multiple modelling systems. This puts interoperability and flexibility at the centre of building tools and resources. As a consequence, systems biology standards and resources must aim for improved interoperability.

The international COMBINE initiative, with its standards SBML, CellML, SBGN, SED-ML and others ( Hucka *et al.*, 2015; Waltemath *et al.*, 2020), has developed a range of interoperability standards for systems biology models, their visualisation, combination, and execution. COMBINE standards form the basis of model resources like the ELIXIR deposition database BioModels, as well as JWS online ( Peters *et al.*, 2017). The ELIXIR Core Data Resource BRENDA, and the ELIXIR resource SABIO-RK ( Wittig *et al.*, 2018) both offer export of mainly literature-based enzymology/reaction kinetics data in SBML format. ELIXIR has strong links to COMBINE, enabling and embodying information exchange between the organisations.

**Challenged interoperability where there are different descriptions of the same or related data.** When analysing data one often stumbles upon the problem that different data models still yield different descriptions, even though both descriptions are FAIR; there is more than one way for a model to be FAIR. FAIR identifiers from different databases, such as those provided by BridgeDb and MetaNetX), ontology terms from different domain ontologies (e.g. OxO versus meta-ontologies like EFO) and connections between different levels of precision in chemical (sub)structures and chemical names (e.g. the ChEBI ontology, the Chemistry Development Kit ( Willighagen *et al.*, 2017), and the various chemistry resources provided by the ELIXIR-CZ Node) should become interoperable with one another. These needs are a challenge not just for systems biology but in fact for ELIXIR, and especially the Interoperability Platform, as a whole, we will need to add the resources needed for interoperability as part of modelling and analysis to the initial work on FAIR descriptions.

The FAIRDOM-SEEK ( Wolstencroft *et al.*, 2015) project data-management system for systems biology emphasizes integration of data and models. It supports researchers and collaborative projects to catalogue, organize, share, interlink and publish local and remote data files, models, protocols, workflows, etc., enabling in particular linking models and their supporting data. FAIRDOM-SEEK’s ‘Search’ includes an external search in the BioModels repository. It integrates the BiVeS tool for describing differences between model versions ( Scharm *et al.*, 2016). Integration of the tools JWS online and COPASI ( Mendes *et al.*, 2009) enables users to run simulations of SBML models directly in the system. Integration with e.g. NeLS ( Tekle *et al.*, 2018) and the Swiss openBIS ( Barillari *et al.*, 2016) data management systems aim at bringing more ELIXIR data close to the models.

**Using descriptive models and linking them to predictive models.** An ongoing challenge of modelling is the linking of standard models with models expressed in scientific programming and general-purpose languages such as Matlab, Python or Julia. Researchers argue that innovative types of modelling precede standardisation, e.g. in the case of languages for exchange of multicellular agent models. Building bridges between different types of model specifications appears a worthy challenge for a network as wide as ELIXIR.

The integrative systems biology community has developed various resources for descriptive models (primarily molecular pathways), e.g. in Reactome, KEGG ( Kanehisa *et al.*, 2008), MetaCyc ( Caspi *et al.*, 2020), and WikiPathways ( Martens *et al.*, 2021). Some early attempts to harmonise the content of these built on the BioPAX standard ( Demir *et al.*, 2010) or simply on gene lists (e.g. Pathway Commons ( Rodchenkov *et al.*, 2020) and the Molecular Signatures Database, MSigDb, at the Broad Institute ( Subramanian *et al.*, 2005)). More recently, dedicated converters allow translation between Reactome, WikiPathways, and the Disease Map resource MINERVA ( Gawron *et al.*, 2016). This supports integrated analysis using the various resources including the conversion of these pathways into biological networks and exploration with network biology tools like Cytoscape ( Shannon *et al.*, 2003). Such networks, combined with experimental data, uncover relevant aspects of molecular biology, like strongly connected parts or strongly regulated parts in the biological system. They also enable linking with gene regulation databases (e.g. transcription factor and microRNA target linking databases) and with databases of chemical interactions with molecular biological targets (e.g. drug-target or molecular toxicology databases). Moreover, such networks can be mapped to biological ontologies (e.g. from Gene Ontology ( The Gene Ontology Consortium, 2019)), disease-related genes (e.g. from OMIM ( Amberger *et al.*, 2019)), and variants (known from e.g. dbSNP ( Sherry *et al.*, 2001) or observed experimentally). Of course, the possibility of linking multiple resources creates new standardisation and interoperability challenges (cf section on Strengthening standardisation & interoperability).

A new development is the link from descriptive models to predictive models in SBML. Since pathway models already support standard descriptions of reactions (e.g. in SBGN and MIM) it is possible to convert them into SBML and that again allows their use as predictive models. This development was recently catalysed by the coronavirus disease 2019 (COVID-19) Disease Map project ( Ostaszewski *et al.*, 2021) that is also supported by the FAIRDOM initiative with its FAIRDOMHub platform to share models and corresponding data. **The basic infrastructure now exists and needs to be further developed, tested, and disseminated (e.g. as training modules).** In essence, this aspect of model interoperability can form the bridge between biological data analysts and predictive modellers working on the same biological systems. This idea was also the driver for the recent fluxomics Implementation Study of the Metabolomics Community. Fluxes are what is predicted by Flux Balance modellers while concentrations of gene products and metabolites are what is usually determined experimentally; the latter are at best a proxy of the former. Measuring fluxes or extending to dynamic modelling, provides the extra linkage.

The MetaNetX reconciliation ( Moretti *et al.*, 2021) of metabolites and reactions aims at providing cross-references between major public resources for metabolism (e.g. KEGG, RHEA, CHEBI) and genome-scale metabolic maps published by different groups (e.g. BiGG, Metabolic Atlas); only such a reconciliation can lead to true genome-scale metabolic models. The computation of the reconciliation considers three lines of evidence: the detailed metabolite chemistry, the description of gene-protein-reaction complexes inclusive of their kinetics, and the dynamic properties of the models, i.e. the distribution of permissible fluxes. Discrepancies, imprecisions, and mistakes in the metabolite chemistry are detected, possibly corrected and converted into cross-references that preserve at best the dynamic properties of the models. The seamless integration of existing models, and their improvement, require more software development and dedicated (application-specific) databases and are in line with the requirements promulgated by the FAIR data consortium.

### Modelling as a service

Models generated via a systems biology approach should be concrete enough for their newly proposed molecular network mechanisms to be validated/invalidated experimentally. This may in part be automated ( King *et al.*, 2009) at the high-throughput level, enabling advanced systems biology research to formulate the crucial questions for understanding the system under study. We expect this to enable a new type of bioengineering, which will develop new production processes as well as more effective medical therapies. The role of ELIXIR in supporting this encompasses the provision of sophisticated data resources and tools and rich standards that are useful both for data mining and experimental design.

Although we may anticipate the entire automatisation of model building in the foreseeable future, for the time being we still need a hand-crafted approach, where every model is unique and personalised for a certain customer. ISBE developed a concept of so-called M4 (Make Me My Model) service ( Kolodkin *et al.*, 2018). The M4 service was provided to various customers. For example, within the framework of the CORBEL project, ISBE has built a PBPK (Physiologically Based PharmacoKinetic) model for a customer from the Environmental Engineering Laboratory in Spain ( Sharma *et al.*, 2020). The experience at M4 service highlighted that every new model requires a new configuration of the modellers team with experts from various modelling approaches. Taking advantage of a large research infrastructure where a flexible team of experts from different modelling areas could be quickly assembled for the needs of a specific project would be very advantageous in this sense.

Another advantage of using a large infrastructure and interoperable platforms is to minimise the need for new models to be built *de novo*. Already existing models for similar systems could be used as a starting point. We should also notice that, due to the similarities in the biological organisation, and as the same building blocks (biomolecules) and similar biochemical processes are used in all organisms, a common blueprint model could be used. Every new organism and every new case will be instantiations of this more generic model. Along with the development of ‘Silicon cells’ or ‘Digital Twins’ of Biological Systems (discussed in section on Strengthening standardisation & interoperability), Models as a Service can become customer-specific tailoring of the already available blueprint model.

These various efforts are, altogether, necessary to make systems biology a true enabler of advancements in a variety of fields, from understanding host-microbiome interactions, to the development of comprehensive ecosystem models, to biobased production processes, to systems medicine. In the following section we exemplify how systems biology plays a crucial role in enabling systems medicine.

### Systems biology underpinning systems medicine

Systems medicine is an implementation of systems biology in the areas of clinical research and practice. It employs computational, statistical and mathematical multiscale analysis and modelling methods to study disease mechanisms towards improved diagnosis, prevention and treatment. The framework of systems medicine is closely related to the concepts of personalised or precision medicine, where the systems approach informs decisions about tailored actions to improve the health of individual patients or patient subgroups. Numerous publications in high-profile journals have confirmed the benefits of systems medicine approaches in promoting precision in diagnostics and personalised therapies in cancer and other diseases, both rare and common.

**Figure 2.**
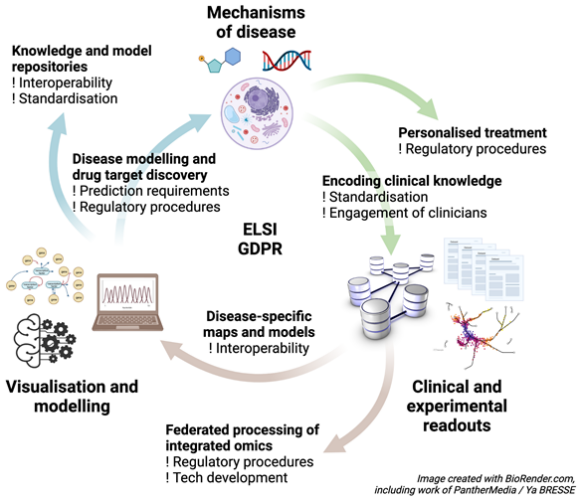
Illustration of the key areas of systems medicine (building on Figure 1). Encoding clinical knowledge; federated processing of integrated omics data; construction of disease-specific maps; construction of knowledge and model repositories in the area; and disease-specific modelling and drug target discovery leading to the development of personalised treatments based on models of disease mechanisms. Underpinning all of these is the need to work within ELSI and GDPR regulations.

Systems biology studies of human disease have to encompass additional levels of complexity compared to other implementations in systems biology ( Apostolopoulos *et al.*, 2020; Wolkenhauer, 2020; Zanin *et al.*, 2021), as highlighted in Figure 2. On one hand, human diseases are phenotypically and mechanistically better understood than diseases of other organisms, but on the other hand, there are difficulties studying these molecular mechanisms in accordance with ELSA and GDPR rules. Additionally, whereas we tend to ignore individual differences when we study model organisms, we do recognise the importance of individual differences in humans, obliging systems medicine tools and approaches to personalise disease interventions. Further, disease therapies, which are themselves complex, are also important areas of study and modelling. Implementations in the broad domain of systems medicine can range from semantic representations of diseases and disease maps through mathematical modelling of diseases to applications aimed at supporting P4 (predictive, preventative, personalised, participatory) medicine and at managing individuals’ health, including linkage to clinical monitoring devices and the use of big data and AI. An important requirement for clinical applications in the future will be the link to personal health data records.

Challenges associated with building personalised systems medicine models are: identification and modelling of network structures that are prognostic and predictive; integration with related systems such as microbes or expososomes; personalising models using patient data; and clinically validating these models.

Solving these challenges will require access to well-annotated patient data, developing standards for personalising and validating models, and a dedicated repository to exchange these models. Importantly, building models fit for clinical use in precision and personalised medicine should start with a clinical problem, and requires close collaboration between modellers and clinicians. An important aspect to addressing these challenges will be linking maps and models to sensitive data held in the FEGA ( Federated EGA), potentially making use of Beacons to identify relevant information (e.g. variation associated with relevant phenotypes). This will become increasingly important with the availability of data from the 1+MG project ^2^. Beyond this, it will be important to improve interoperability of models with other data sources and tools. ELIXIR can help address the challenges of linking models to clinical information through the work of its Health Data Focus Group. A general technical issue, shared by systems medicine and other applications that will make use of sensitive data in the human health and genomics domain, will be the development of federated learning algorithms that are able to learn models based on data held on a number of restricted-access servers. Furthermore, standards and solutions will need to be developed to link dispersed health information to integrating models such as disease maps and digital twins. At the same time, it will be important to improve interoperability between maps, models and digital twins and ELIXIR’s Core Data Resources and Deposition Databases.

At the core of systems medicine lies the involvement of the user communities: the clinicians and the patients. The patient-oriented approach with the integration of personalised data, both clinical and omics, into network-based analyses and models will enable tailored stratification, therapies, disease management strategies and monitoring. A key element that systems medicine can bring to ELIXIR is its engagement with clinical researchers collecting disease-relevant data and the application of its approaches close to the clinic.

Visual exploration and analytics of computable disease models, which bridges the expertise of clinical experts and the methods of bioinformaticians, enables knowledge about molecular disease mechanisms and relevant clinical data to be brought together for meaningful interpretation, thereby reducing the complexity of the knowledge and the scale of data ( Satagopam *et al.*, 2016). This is greatly aided by disease maps ( Mazein *et al.*, 2018), an emerging methodology for building human and machine-readable models of molecular disease mechanisms. They offer online and interactive exploration of diagrams describing molecular and cellular hallmarks of different disorders, with detailed annotations of participating molecules, and citations of articles describing the encoded mechanisms.

Construction of these maps is a challenge, as it requires close interactions with clinical experts, continuous quality checking against emerging facts and data, and persistent evaluation to support downstream modelling approaches. ELIXIR is in a prime position to support building such visual and computable repositories via its newly established Disease Maps Node Service and by engaging relevant communities, e.g. Rare Diseases, Federated Human Data, 3D-Bioinfo, Metabolomics, Proteomics, Galaxy, and others, many of which themselves engage in visualisation and modelling of different kinds of biological entities.

An example of such an ecosystem supported by ELIXIR is the COVID-19 Disease Map, engaging clinicians, life scientists and computational biologists to set up a graphical and computable repository of severe acute respiratory syndrome coronavirus 2 (SARS-CoV-2) mechanisms. Engagement of a highly motivated community has resulted in an interoperable repository of curated diagrams following systems biology standards ( Schreiber *et al.*, 2020), integrated with interaction databases and text mining platforms. We can consider this effort as a blueprint for building qualitative systems medicine models, which will feed downstream, detailed modelling workflows ( Fröhlich *et al.*, 2018). A future aim for systems medicine within the Systems Biology Community will be to facilitate the construction of disease maps for currently unrepresented disease areas.

Incorporating individual differences into systems biology models can be used to create personalised models akin to a “digital twin” of a patient with a disease ( Fey *et al.*, 2015). Modern omics technologies, such as genome sequencing, allow molecular profiling of individual patients, with unprecedented resolution down to single cells. A prominent example is *The Cancer Genome Atlas*, an enormous repository of multi-omics data from over 11,000 cases across 33 cancer types ( Hutter & Zenklusen, 2018). Populating systems biological models with personal data can yield highly individualised models that can help simulate disease evolution and response to therapy with high sensitivity and specificity ( Barrette *et al.*, 2018; Béal *et al.*, 2021; Bhinder & Elemento, 2017; Crawford *et al.*, 2018; Eduati *et al.*, 2020; Fey *et al.*, 2015; Hastings *et al.*, 2020). These systems medicine models are knowledge-based and thus able to offer insight into the disease and drug response mechanisms of a patient ( Hutter & Zenklusen, 2018; Ebata *et al.*, 2022). For example, a personalised model of the JNK stress-response network resulted in refined patient-stratification for neuroblastoma, a common childhood cancer, and revealed an impairment of the JNK apoptotic switch in high-risk cases ( Fey *et al.*, 2015). As more and more clinically-validated models of this kind arise we foresee a need for a repository for them which might, for example, be built in conjunction with Reactome and the proposed ModeleXchange initiative (see the Strengthening standardisation & interoperability section below).

Personalised medicine, and therefore systems medicine, is an active area of industrial development in Europe ( European Biopharmaceutical Enterprises, 2015). Facilitating linkage of systems medicine into the ELIXIR ecosystem of data, tools and standards can therefore be expected to have an impact on the development of this sector in the future.

A need that systems medicine shares with other applications in the Human Data domain is to address ethical challenges of (i) accessing data for constructing models, (ii) the use of models and (iii) the use of their outputs. For example, constructing models using a combination of datasets describing an individual might increase their identifiability and aspects such as data ownership and the ability to withdraw consent from datasets could affect the persistence of models. The Community will therefore engage with ELSI efforts in the Human Data Communities, within ELIXIR and beyond, to investigate, for example, the impact of ELSI issues on building, constructing and sharing models based on human data. Close collaboration is also envisioned with the Genome of Europe initiative, which will start by collecting ELSI compliant human datasets from the general European population. Another ELSI aspect is engagement with industry, to ensure ethically acceptable uptake of the techniques and infrastructures it develops from lab to the bedside. Finally, the Community will work on the dissemination and outreach of developments resulting from the Community’s work to clinicians and patients.

### Capacity building and training

The stark rise of high-throughput technologies and related datasets describing the complexity of biological systems in health and disease, has been a driver of the emergence of systems biology, which comes with inherent training challenges. The ELIXIR Systems Biology Community has identified several challenges that need to be addressed for developing and delivering successful systems biology training that could be achieved within different time frames. These are detailed below.

**Figure 3.**
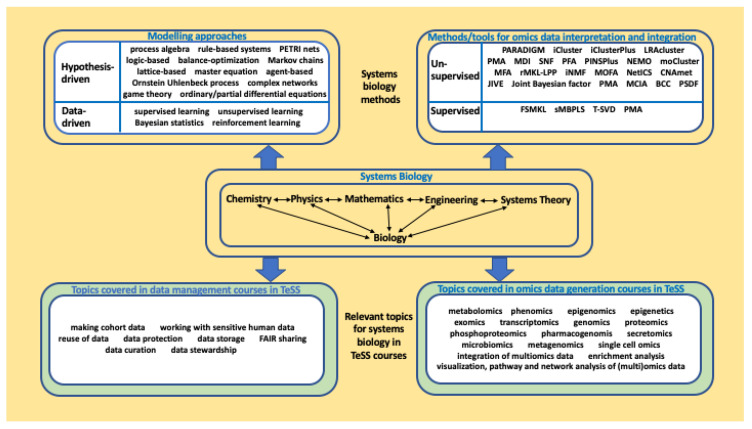
Training courses available in TeSS can be fully integrated under the systems biology umbrella. Methods/tools for omics data interpretation and integration are categorised based on unsupervised/supervised approaches ( Subramanian *et al.*, 2020).

***Diversity in trainee backgrounds.*** Owing to the diversity of systems biology approaches, heterogeneity of trainee backgrounds is common. Trainees often differ greatly in their knowledge of biology and mathematical modelling, as illustrated in Figure 3, and their levels of experience with software tools or systems biology methods also varies from ‘novice’, to ‘competent practitioner’ and ‘expert’ user. Finding the optimal balance between providing sufficient information on systems biology databases and software and not overloading trainees with too many new concepts, represents one of the main training challenges.

To accommodate different trainee backgrounds, an option might be to pre-screen trainees well-ahead of the training event to understand their expectations of what they will achieve at the event. This could be in the form of a questionnaire, hosted by TeSS ( Beard *et al.*, 2020) or sent out on a mailing list, with the objective to (1) assess the trainee’s abilities, (2) find out their training needs, and (3) make recommendations on existing courses based on (1) and (2). This may prove a good opportunity to synergize training with other biomedical communities (e.g. fluxomics, microbiomics) and offer general bioinformatics courses (e.g. on reproducibility and data management, omics data analysis). Such information will not only help the trainers to adjust the course material, but also establish uniform criteria for pre-requisites of the course. Building a systems biology concept map covering the basic areas of systems biology research and perspectives should be of value. The organisation of hackathons to decide on the concepts that are to be included in more specialised training courses depending on the level of maturity of the trainees in systems biology, has proven to be a successful practice for setting up an ELIXIR training school in a rather diverse area of expertise.

***Creation of a systems biology learning path and broad promotion of the integrated systems biology framework.*** Although some independent ELIXIR training activities already deliver systems biology-related training modules (e.g. single-cell omics, metabolic modelling, data integration), many may well be missing or unknown. There is a need for integrating new systems biology courses into TeSS and for integrating them with existing courses under the systems biology umbrella.

In line with the efforts of the ELIXIR Training Platform in Task 2, the ELIXIR Systems Biology Community will define a learning path, identify gaps on missing topics and suggest suitable courses. For example, there may be a need for a course on reproducibility of systems biology models, on handling sensitive human data, or on specialised omics topics. As depicted by Figure 3, the systems biology framework can be well-complemented by omics data generation courses and data management courses, already covered in TeSS.

Coordinating educational events across borders is an important part of training. The community will engage in facilitating the collaboration of ELIXIR Nodes for training purposes. Additionally, the ELIXIR Systems Biology Community will initiate joint events with other ELIXIR communities via workshops and hackathons. A complementary approach may be to collaborate with non-profit organisations in the advancement of systems biology-related areas and education, such as iGEM. 

We will systematically review the interest in novel systems biology topics and will propose new courses, based on demand. Dissemination of existing (see Table 3) and new systems biology courses will be enabled by the TeSS platform, with some of the systems biology-related ELIXIR training courses already reported in TeSS. New systems biology courses will be integrated into TeSS and further promoted by organising ELIXIR-level international courses. To reduce the technical issues associated with broken web links on TeSS, we will perform regular checks of these and will also encourage our trainees to report any issues to the site’s administrator. Moreover, we will investigate the overlap between TeSS and other training resources such as the Galaxy Training Network. E-learning approaches for courses and training educational resources will be incorporated in collaboration with the Training Platform and its services.

**Table 3.** Examples of systems biology courses across the ELIXIR Nodes.

Course	ELIXIR Nodes involved	Length of the course	Training material publicly available
Systems biology: From large datasets to biological insight	EMBL-EBI	5 days	yes
Mathematics of life: Modelling molecular mechanisms	EMBL-EBI	5 days	no
ELIXIR Omics Integration and Systems Biology	ELIXIR-SE	5 days	yes
Course portfolio Bioinformatics and Systems Biology	ELIXIR-NL	5 days	no
Tools for Systems Biology modelling and data exchange: COPASI, CellNetAnalyzer, SABIO-RK, FAIRDOMHub/SEEK	ELIXIR-DE	3 days	no
Computational Systems Biology for Complex Human Disease: from static to dynamic representations of disease mechanisms	ELIXIR-FR, ELIXIR-UK	5 days	yes
ELIXIR Fluxomics Training School	ELIXIR-GR, ELIXIR-ES	5 days	partially
Hands-on tutorial Systems Biology/Medicine	ELIXIR-SI	3 days	no

***Availability and use of standardised datasets in training materials.*** With the ever-changing systems biology landscape, new tools and massive amounts of data become available and so, trainers need to continuously adapt to these developments. One of the training challenges relates to the lack of standardised and FAIR datasets underpinning specific topics and the lack of suitable documentation for some of the tools or databases. This makes it difficult for trainers to keep their materials up-to-date. In addition, training materials are often focused on specific software or databases, rather than providing an overview of a given topic.

Sharing real or synthetic datasets could prove especially useful, for example, as a part of practical hands-on exercises or use cases. The ELIXIR Systems Biology Community will aim to (1) promote the use of appropriate data sets for testing and/or validation in different omics areas, underpinned by ELIXIR Core Data Resources such as The Human Protein Atlas ( Uhlen *et al.*, 2010) or MetaboLights ( Haug *et al.*, 2020), (2) provide a web-based platform for self-education via online tutorials, (3) establish a network of regular trainers and invited speakers, in order to readily communicate best practices and share materials.

***To provide support to trainers, in order to build a variety of training expertise that can meet the demands of this fast-growing field and the training needs of its users.*** Supporting the needs of current and future trainers is a key objective of the ELIXIR Systems Biology Community. Identifying specialised trainers from the TeSS network and creating a centralised repository of training materials, would help enormously with this task. In addition, joining the train-the-trainer events organised by ELIXIR will help improve the quality of training.

There is a lack of teaching courses that would be designed for trainees who wish to learn particular systems biology tools or methods in order to train others. The ELIXIR Systems Biology Community will facilitate the delivery of courses with the aim of (1) training trainees on how to use systems biology resources and (2) providing them with good training material to disseminate the knowledge acquired during the course. The network of trainers will have access to a centralised repository of systems biology training materials, hosted by TeSS, which could save trainers a large amount of material preparation time. Such repositories may contain downloadable PowerPoint slides, lesson plans, high-resolution images in editable format, a list of web links to systems biology resources related to training, ranked based on relevance to a given topic, and models that can be run at a click. This may sound ambitious, but once created, the resources can be updated (e.g. replacing a tool with a newer one).

### Industrial embedding

Systems biology is a multidisciplinary endeavour to gain insights into the complexity of biological systems, pervasive to all colours of biotechnology ( Kafarski, 2012) ^3^. It is, thus, also bound to play a major role in the translation of this knowledge into applications of industrial, medical, agricultural and environmental interest. However, the systematic deployment of systems biology varies greatly across sectors, and its potential often remains insufficiently exploited. In this subsection, we map some of the major embeddings of systems biology in various industrial sectors and pinpoint key challenges.

Various biotechnological and pharmaceutical companies started their own systems biology programs years ago and stimulated academic parallels. Already in the 2000’s, AstraZeneca instated a research chair in systems biology at the University of Manchester, UK, which then became instrumental in setting up largely academic research and doctoral training centres at that same and at other UK universities. In parallel, AstraZeneca and Pfizer developed and published the first systems biology models of signal transduction, relevant for the targeting of anticancer drugs. Bayer spun out a smaller company focusing on systems biology and data analysis. These initiatives also led to systems biology penetrating adjacent fields and setting up Systems Medicine, systems pharmacology, and systems toxicology.

The EU’s Innovative Medicines Initiative (IMI), co-funded by industry, brought the necessity of these new disciplines to the fore. Many of the supported IMI projects lacked systematic long-term stability, owing to the short innovation life cycle in the pharmaceutical industry. Initiatives of this kind would benefit from long-term sustenance by a European research infrastructure. TransQST, an IMI-funded consortium with industrial-academic partnership with links to ELIXIR’s Toxicology Community, aims to build novel systems toxicology models that enable translation from non-clinical to human safety during clinical trials. Quantitative data and resources generated within this initiative are sustainably disseminated through ELIXIR Core Data Resources, such as ChEMBL ( Gaulton *et al.*, 2017), ArrayExpress ( Athar *et al.*, 2019), and deposition repositories, including BioStudies ( Sarkans *et al.*, 2018) and BioModels.

PK-PD (pharmacokinetics-pharmacodynamics) modelling is an essential component of the drug discovery and development pipeline, which involves mathematical approaches to study pharmacokinetics (PK), pharmacodynamics (PD), and their relationship ( Danhof *et al.*, 2005; Peck *et al.*, 1992). The process of drug absorption and disposition by the body is defined using quantitative PK models. Pharmacological effects of a drug on the body taking into account the mechanism of drug action are quantitatively described using PD modelling. The existing approach is however void of biochemical detail and cross-fertilisation with systems biology has been leading to Physiologically-Based PK (PBPK). This may address the roles of active drug efflux pumps as well as kinetic details of drug metabolism in the liver by P450s [e.g. Sharma *et al.*, 2020]. PBPK modelling is also starting to be explored as a key element of a safe-by-design approach for material development (for instance in the DIAGONAL EU project for evaluation of nanomaterials). The European Medicines Agency (EMA) has stipulated guidelines on qualification and reporting of PBPK analysis ( Zhao, 2017).

Quantitative Systems Pharmacology (QSP) is a converging point of biochemical pathway analyses and pharmacological modelling, and falls under the broader umbrella of systems biology. Much of such work has been funded by the IMI. The UK-QSP network with the UK and international scientists in industry and academia is jointly funded by the Engineering and UK Engineering and Physical Sciences Research Council (EPSRC) and Medical Research Council (MRC) with financial assistance from AstraZeneca, Pfizer and GlaxoSmithKline.

There is a strong parallel between systems biology approaches described above for pharmacology and those used in toxicology. The same kinetics ("does it get there?") and dynamics ("what does it do?") modelling approaches apply. Mechanistic models used in toxicology like adverse outcome models and quantitative approaches for read-across, predicting endpoint information for one substance by using data from the same endpoint from (an)other substance(s), also have their counterparts in pharmacology.

Bioinformatics approaches towards mining literature and databases for comprehensive metabolic data have led to the development of maps based on genome-scale metabolic models (GEMs) for a multitude of organisms, including humans ( Robinson *et al.*, 2020; Thiele *et al.*, 2013). Constraint-based modelling (‘FBA’ and derivatives) of these GEMs is a systems biology product that enables the understanding of a variety of processes such as new metabolic ramifications of tumours ( Damiani *et al.*, 2017).

Analogous applications to microorganisms have led to multiple new insights into how these organisms may be engineered towards higher productivity ( Wehrs *et al.*, 2019). These types of activity have, at the European level, been linked with industry in various settings, including explicitly the ERA-net CoBioTech Action and implicitly the Biobased Industries Joint Undertaking [and its successor] promoting systems biology and synthetic biology as technology drivers to speed up research and innovation in industrial biotechnology. This is particularly relevant when addressing key priorities of the European Union with regards to a green transition to a biobased, environmentally sustainable economy.

The bio-economy comprises the production and use of renewable resources from land and sea, and the use of waste to make value-added products, such as food, feed, bio-based products and bioenergy. In the EU, the bio-economy is worth an estimated €2 trillion, employing 9% of the workforce. It provides a unique opportunity for Europe to develop sustainable processes and address grand challenges, such as tackling the effects of climate change, achieving a clean environment, fostering industrial innovation, tailor-producing chemicals, contributing to healthy living and ensuring food security. Among the ways to address these challenges, the use of microbial organisms offers a valuable and powerful alternative to the fossil fuel-based economy as they can be engineered into cell factories producing fuels, high-value compounds, chemical building blocks, nutraceuticals and novel medicines (see bioconsortium.eu and the ELIXIR Microbial Biotechnology Community). This is best done through synthetic biology, which is frequently defined as the application of engineering principles to biology. Such principles [model-driven design, modularization, standardisation, separation of design and fabrication] enable streamlining the practice of biological engineering, to shorten the time required to Design, Build, Test and Learn (DBTL) biological systems. This streamlining of iterative design cycles facilitates the construction of more robust microbes that are better adapted to the target application and behave in a more predictable fashion. The streamlining benefits also hold for the engineering of consortia of interacting microorganisms, which allows to explore the wealth of microbial diversity. Furthermore, the individual processes have to be tackled by taking into account the whole value chain if they are to result in economically feasible innovations which can truly contribute to a shift from a petrochemical to a biobased economy ( Kampers *et al.*, 2022). ***The principles, methods and (data and model) resources underpinning systems biology are crucial to reach these objectives***. Over the last 5 years, there has been a substantial increase in the number and variety of companies that both adopt and build upon these principles and technologies for their business development. A few examples include DSM, Lanzatech, Zymergen, Ginko Bioworks, Amyris, and Genomatica and, recently, a major push has been made by the USA government on this with substantial investment ^4^. Crucial to these developments is the increasing focus on advanced, (semi-)automated infrastructures that enable rapid, tailored biomanufacturing of chemicals and materials, such as those by ESFRI programme IBISBA ^5^ and other biofoundries ^6^.

The same basic principles and many methodologies are relevant when tackling challenges in agriculture, in particular in plant and animal sciences, as well as in ecology and water and soil management, and, crucially, in addressing climate change issues, such as reduction of emissions at source (see industrial biotechnology above), carbon capture and nutrient recycling.

## Systems Biology within ELIXIR

**Figure 4.**
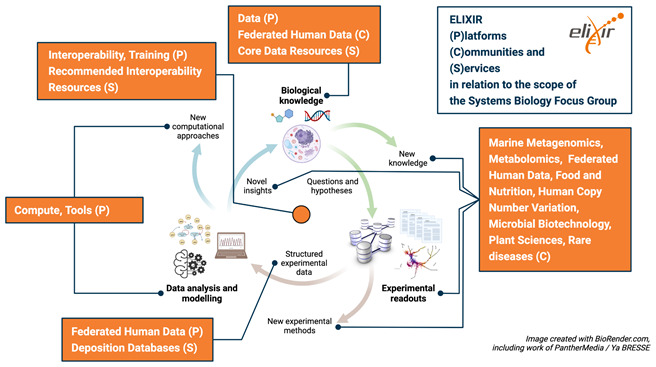
Schematic depiction of the systems biology “cycle”, mapped to ELIXIR Platforms, Communities and Services. This illustration shows the central role systems biology may play within the life sciences and how it links to and can supplement existing ELIXIR activities. The scheme spirals out of the plane of the paper in the sense that biological knowledge, experimental data, and models all increase in quality with each turn of the cycle.

**The infrastructure aspects of systems biology and systems medicine - databases, tools and standards development as well as training and access to cloud infrastructure - are not only appropriate components of the ELIXIR infrastructure, but will be essential for ELIXIR’s future support of advanced biological applications and personalised medicine (see Figure 4).**

Because in systems biology experimental data relate to emergence of function through computational models, and because computational models need to be based on realistic experimental data, integrated data-and-model repositories such as *JWS Online, BioModels* and *Metabolic Atlas* are essential. Models and data need to be accessible to external users, for curation, validation and critical evaluation: the model-data repositories need to be ‘live’ in that they readily simulate the spatial-temporal behaviour of living systems. They should enable ‘what-if’ computational experiments *in silico*, in order to serve bioengineering, medicine, pharmacology and basic biology. Further, these repositories should allow for constant refinement and update of the models that they host. We can build on the landscape of data resources that ELIXIR already has in the form of Core Data Resources for knowledge used to build the models, and Deposition Databases for parameter tuning, validation, etc.

Standardised model annotation of the described components such as genes, reactions, or metabolites is required to ensure interoperability between models and FAIR repositories hosting large omics datasets. While extensive guidelines and test suites for such annotation have been developed in the COMBINE context and MEMOTE ( Lieven *et al.*, 2020), respectively, their evolution and consistent application would benefit from a strong infrastructure context.

In relation to ELIXIR, the Systems Biology Community will enable a wide range of researchers to benefit from existing and proven systems biology and bioinformatics approaches, rather than to reinvent wheels. A number of applied Communities in ELIXIR, such as Rare Diseases, Food and Nutrition and Toxicology, form bridges to large research fields. We offer to support these ELIXIR Communities with the standards and best practices of the systems biology ‘ecosystem’ and build on ELIXIR Platforms to enable better understanding and reproducible analysis of complex systems.

### Synergies with ELIXIR Platforms

ELIXIR Communities and Platforms cover topics, including:

- Technology, like the Galaxy Community and Bioschemas (and Compute in general);
- Biological entity descriptions, in Core Data Resources, and in Communities like Metabolomics, Proteomics, Copy Number Variation and Intrinsically Disordered Proteins.
- Applied Communities like Rare Diseases, Plant Science, Food and Nutrition, and Toxicology.

The Systems Biology Community intends to become the link between these, providing the model ecosystem to the applied Communities, allowing the usage of data from those concerned with biological entities, and using the computational technology approaches and data infrastructure experiences and tools to do so. That linkage again builds on activities in the Interoperability Platform.

There are clear synergies between the objectives and activities of the Systems Biology Community and the ELIXIR Platforms, as described in the following sections.

***The data platform.*** The ELIXIR Core Data Resources and Deposition Databases are already highly used by the systems biology community. For example, data from BRENDA, STRING, and Reactome are essential both for construction of molecular pathways and parameterization of molecular reactions. Furthermore, an essential systems biology database, BioModels, is already part of the ELIXIR Data Platform. With JWS-Online, it facilitates the discovery, deposition, and re-use of systems biology models. SABIO-RK is an ELIXIR Node service which provides curated data manually extracted from literature to modellers. This demonstrates the compatibility of models for data with data for data. Systems biology could contribute new databases to the platform, such as JWS-online, Make Me My Model and many others, which could serve as an example for data service-based resources and databases. Finally, through ELIXIR, the Systems Biology Community, which already features among the largest users of the ELIXIR data resources, should be able to provide support and advice for further development of these resources, so that they become more compatible with predictive, modelling-based knowledge development, making the data more actionable. The engagement of the Data Platform in curation efforts also supports the maintenance of systems biology resources and improves the data quality.

***The tools platform.*** Systems biologists have in the past developed a range of tools, which could find their home within the Tools Platform. Adding systems biology tools to bio.tools, BioContainers ( da Veiga Leprevost *et al.*, 2017) and workflowhub.eu will improve the usage of the already available tools making them better findable and interoperable and applications more reproducible while making it possible to construct new workflows that combine data and modelling operations. Examples of workflows for systems and synthetic biology are embedded in the ESFRI IBISBA. SynBioCAD ( du Lac *et al.*, 2020) is the first Galaxy toolshed for synthetic biology and metabolic engineering. These workflows are registered in workflowhub.eu. It will also be of interest to investigate opportunities to engage with OpenEBench to benchmark models and workflows.

***The compute platform.*** Although specific and large-scale computational simulations require tailor-made, large computational infrastructure, a vast majority of systems biology simulations can be and are executed on general-purpose computational platforms. In the same way that ELIXIR is enabling its members to use national and European compute service providers for their data management needs, it could provide access for systems biology modelling. Through the Compute Platform we will also be able to link to EOSC activities related to Cloud data and workflow execution and also provide convenient access options via the Life Science AAI.

***The interoperability platform.*** Standards are as big an issue in models as in data. Only data and models that use standardized file formats, metadata and vocabularies can truly be FAIR and used by the whole community. The Interoperability Platform’s Recommended Interoperability Resources help to improve this FAIRness while the Platform’s standards mapping resources, such as BridgeDB, identifiers.org and OLS, facilitate better interoperability and integration of data and models. In systems biology, the COMBINE consortium ( Hucka *et al.*, 2015) is the international initiative that coordinates the development of standards, and the use of standards in systems biology is continually increasing. The harmonization between data and model standards, therefore, presents a great opportunity in the next decade, and ELIXIR is in a perfect position to catalyse such a development. A more systematic collaboration with COMBINE should lead to harmonisation of the standards landscape for systems biology. Practical collaboration between the hackathon initiatives from COMBINE, HARMONY, and the Biohackathon can also be expected to be fruitful. Because systems biology combines all the different aspects mentioned above, it is a testbed and impact case for interoperability approaches, further fueling new interoperability developments.

***The training platform.*** The ELIXIR Training Platform remains instrumental in the training of the next generation of data science experts. While there have been plenty of smaller-scale initiatives for systems biology training in Europe at the same time, the overall scale of training in systems biology has been much smaller, mainly caused by a more fragmented systems biology community. To lift-up systems biology training Europe-wide, we propose the joining of the Systems Biology Community and data resources under a single roof. Upscaling training can benefit from FAIRification of training materials, a process which is well supported by the ELIXIR FAIR Training Focus Group. Embedding systems biology training within the ELIXIR Training Platform via TeSS will also enable their integration into Learning Paths which are under development. The Training Platform will help the ELIXIR Community to develop new training materials through training hackathons that will be hosted together with existing training resources in standardised data repositories via TeSS.

### ELIXIR Communities and focus groups

As indicated already in the section “Current Systems Biology activities in ELIXIR” above, systems biology is already a key component of the work of a number of ELIXIR Communities, and it can play an important role in others. Moreover, systems biology also plays a role in various focus groups. Some possible contributions of a future Systems Biology Community to the ELIXIR Communities and Focus Groups are described below in a non-exhaustive manner.

Technology-oriented Communities:

- *Galaxy:* In a recent Community-led Implementation Study, ELIXIR supported the integration of omics data access and analysis tools into Galaxy workflows. Building on this work, an initiative to improve connectivity between Galaxy and systems biology model repositories, development and simulation resources should provide a strong, practical boost to the ELIXIR Community-driven integration between Data and Models for Life.

Biological Entity Communities:

- *Metabolomics*: Aimed towards the chemical, mechanistic and reaction flux-related aspects relevant for biomedical applications, microbial biotechnology, plant sciences, toxicology and nutrition, and the workflows and data interoperability aspects needed for those. Getting from metabolite levels to fluxes strongly links to quantitative dynamic models in systems biology. The Implementation Study “ Standardising the fluxomics workflows” of the Metabolomics Community includes aspects of how ELIXIR could contribute to systems biology methods and workflow standardisation.

Applied Communities:

- *Microbial biotechnology*: Aims to support the computational infrastructure underlying the Design - Build - Test - Learn (DBTL) cycle in the design of industrial microbes. It aims to contribute to addressing standardisation and other issues in relation to models in microbial biotechnology (e.g. semantic ontologies) and thereby contribute to a knowledge-based infrastructure for biotechnology.
- *Plant sciences:* An interdisciplinary group of researchers very active on the border between experimental and computational approaches that aims at building a tools service bundle that will support plant scientists in the integration and linking of diverse datasets with an extension towards systems biology. Many of the current activities of the Community are in the field of experimental data management tools. Expertise from the systems biology community would in the short term enable the extension of these activities to the management of plant systems biology models which are becoming increasingly prominent in the field.
- *Food and nutrition community:* Aims to support the research towards the effects food choice and nutrition have on health and well-being. This typically is a system-wide effect where many small changes work in combination.
- *Toxicology:* Aims to support risk assessment of chemicals, drugs, cosmetics ingredients and nanomaterials to lead to safer products. This includes the combination of toxicokinetic (exposure, uptake, distribution, metabolism) and the toxicodynamic (molecular interactions and complex connected events in adverse outcome pathways). Both aspects and their combination strongly lean on models and modelling.
- *Marine metagenomics (Microbiome)*: Aims to develop a sustainable metagenomics infrastructure to enhance research and industrial innovation within the marine domain. It develops standards and best practices for the marine domain, provides databases specific to marine metagenomics and develops tools and pipelines to enhance metagenomics analyses. These goals will benefit from the standardisation efforts of systems biology elements and, in particular, from the deployment of metagenome scale (metabolic) models.

Human data communities:

- *Federated human data:* Activities in the domain of human data focus on the sharing of human data, predominantly but not exclusively in genomics making use of the increasingly sophisticated Federated EGA and Beacon infrastructures. Although focusing on genomic sequences, FEGA also accommodates phenotypic and disease information which has potential uses in systems biology and systems medicine.
- *Rare diseases:* Implementation Study “ ELIXIR Rare Diseases Infrastructure (2019-21)”, although not explicitly addressing the needs of systems biology or systems medicine, addresses the linking of infrastructures needed to interpret data on rare diseases as well as collecting data that is “FAIR at source”. There is an opportunity to link these objectives to a systems medicine perspective on rare diseases.

ELIXIR also runs a number of focus groups (FGs) that are relevant to the Systems Biology Community:

- *Machine learning (ML):* This focus group was initiated in October 2019 to address needs related to the application of ML in mining large omics datasets to uncover new insights in the field of medicine. These complement activities in systems medicine. Goals of the ML focus group relate to the development of controlled terminology/ontology and services for ML model description. There is an opportunity to align these developments with efforts on standardisation of models in systems biology.
- *EOSC:* Connects ELIXIR’s EOSC-related activities, which run along the axes: a) Consolidation of e-Infrastructure services and positioning these services as an embedded “supply-chain” for data-intensive scientific collaborations; b) Open Science in practice; c) Integrating the user-focussed services from research infrastructures with their user communities to enable interdisciplinary research aligned with the major societal challenges. Systems biology and in particular its focus on model standards, workflows and model-driven activities, can contribute and help to expand the aforementioned EOSC ELIXIR activities.
- *Registries:* identifiers.org, an ELIXIR Interoperability Resource, was founded to fulfil a need of the systems biology community, and now provides a key registry of data resources for both ELIXIR and systems biology. Bio.tools, provides a key registry of tools with strong coverage of ELIXIR-based tools, but has limited coverage in the systems biology domain. Systematic curation of both resources and harmonisation of their computational interfaces, in collaboration with the Galaxy community and in the context of the Interoperability Platform, would provide a strong contribution to the essential integration of models and data.
- *Biocuration:* This group was established in 2021 and builds a network of database developers and curators in ELIXIR. The Systems Biology Community benefits from the interaction with the International Society for Biocuration and the engagement for a better visibility and recognition of the work of biocurations, e.g. community curation efforts in model resources.
- *FAIR training:* The FAIR Training FG was formed in 2018 with the aim of improving the production and diffusion of FAIR training materials across Nodes. These activities closely complement those of the Systems Biology Community whose applications often rely on multi-omic and holistic datasets and respective descriptors and identifiers for effective delivery of materials and tools of systems biology.
- *Biodiversity:* This group is focused on understanding and cataloguing the capabilities, interests and ongoing projects we have in this area across the ELIXIR Nodes. It develops appropriate connections with key external partners in the field. Large-scale systems biology models of interactions and evolution are bound to strengthen the area, as exemplified by various specific research projects across Europe

## The Community’s objectives

To illustrate the importance of infrastructure for systems biology in a forward-looking way, we have identified a number of potential challenges for the Systems Biology Community that would rely strongly on the infrastructure ELIXIR provides. These challenges align with those of other ELIXIR Communities, as outlined previously in this paper. They sit on a scale comparable to the grand challenges that ELIXIR tackles in its programme, namely:

- To deal with the increasing volume, complexity and heterogeneity of data,
- To enable the interoperability between data resources,
- To effectively use large, complex and heterogeneous data sets to generate actionable knowledge,
- To make it easier to find and deploy the right tools and to undergo training,
- To build data interpretation and modelling infrastructure following FAIR principles,
- To drive innovation and industry usage.

A major remit of systems biology is to quantitatively describe the dynamic, emergent interactions among the many components of biological systems. The goals are hence the generation of insights and knowledge, which can be translated into applications of industrial, environmental, nutritional, medical, ecological, and agricultural interest. For instance, predictive dynamic models of cells, organs and organisms have a potential for pre-testing drugs, producing new materials and chemicals for food & feed, understanding biogeochemical cycles, tackling carbon storage, and accelerating the shift from a petrochemical to bio-based economy. Models are crucial to understanding and fostering human and animal nutrition, host-microbiome interactions (plants, insects, animals, environment) and a range of other areas. These include the biochemical brain; systems ecology, agriculture and environment; individualised medicine enabling the prediction of the effects of 2,4-Dinitrophenol (DNP) in physiology and pathology; systems pharmacology enabling the individualised prediction of drugs’ effects and toxicity, as well as model-driven production of tailored pharmaceuticals; systems epidemiology enabling the critical prediction of how government and therapeutic measures affecting pandemics such as that of COVID-19.

All these areas will benefit substantially from systematic and comprehensive bioinformatics and mechanistic and realistic modelling. Below we lay down five pillars around which this ELIXIR community aims to contribute to strengthen systems biology.

### Strengthening standardisation & interoperability

The standardisation needs in systems biology remain diverse (see Table 4). Data and models, as well as metadata of both, need consistent structuring following standardised formats. Close collaboration of ELIXIR with standardisation communities dedicated to modelling in the life sciences, such as COMBINE, as well as with relevant committees of standardisation bodies like CEN/CENELEC and ISO, such as the ISO committee for biotechnology standards ( ISO/TC 276) with its working group WG5 “Data Processing and Integration” or the ISO committee for health informatics ( ISO/TC 215), will ensure further development and adaptation of existing modelling standards to the needs of models shared via ELIXIR resources. Based on modelling standards like the ones from COMBINE ( Golebiewski, 2019), model validation becomes more realistic. An advancement in this direction has been the release of the standardised genome-scale metabolic model testing tool MEMOTE. Retrospectively applying this tool to existing models, however, remains an open challenge. We will seek to contribute to the standardisation and interoperability of data, operations and models.

**Table 4.** Breakdown of short, mid and long term objectives for the Standardisation & Interoperability theme.

	Aims and objectives
**Short term (~3 years)**	• Better support of existing standards in model repositories • Build upon systems biology models to improve the design of experiments that lead to the generation of higher quality, quantitative, FAIR data; • Address specific challenges for human modelling, which include: ○ working with compartments; ○ model validation through standardised phenotypes; ○ initial interfaces for multi-level modelling and integration across scales; multi-tissue evaluations; ○ extrapolations from single cell analysis to tissue level; ○ microbiome - host interactions; ○ integrating sensitive personal data into models for personalised medicine • Establish approaches for model exchange, building on existing resource developments in the FAIR data landscape (e.g. FAIR data points; JWS-Online), BioModels and ModeleXchange. • Improved interoperability of modelling, simulation and analysis tools
**Mid term (~6 years)**	• Improved standardisation of generation of complex data incl time-series, functional and imaging data for integration in computational models • Providing a link between existing models and datasets for easy access to relevant data • Good strategies (including training) to improve reproducibility, credibility, and validation of models and to assess the efficiency of tools, leading to the development of quality marks, thereby increasing the quality of workflow outcomes
**Long term (~10 years)**	• Improved interoperability of data and models to enable FAIR model connection and integration (at different scales) so as to facilitate the development of multi-scale modelling frameworks;

**Modelling repositories.** Current work across ELIXIR Nodes has begun to partly address these challenges. For example, Metabolic Atlas is promoting the use of a template code repository called *standard-GEM* for open-source genome-scale metabolic models. Conceptually similar to the COMBINE archive, standard-GEM establishes a folder and file format structure that fits with the iterative, versioned model maintenance process. In turn, such a standard structure enables future automatic validation with tools such as the aforementioned MEMOTE, and opens the door towards packaging with RO-Crate ( Soiland-Reyes *et al.*, 2022) and potential integration with BioModels, JWS-Online and OpenEBench ( Capella-Gutierrez *et al.*, 2017) via COMBINE.

Another ELIXIR effort is the established service MetaNetX, which cross-checks model annotation and reports inconsistencies in identifier mapping with respect to chemistry, in addition to facilitating cross-model mapping. Building on this knowledge-base, a potential future direction is the development of a service focused on assessing the quality of the annotation, which would complement the quantitative assessment that is already covered by MEMOTE.

On a wider-reaching level, model repositories like BioModels, FAIRDOMHub and its JWS-Online and others collaborate on the development of community standards, but they are only starting to co-ordinate their curation and dissemination activities. Currently users still need to access multiple repositories to discover all models potentially relevant to them. Moreover, a recent large-scale study ( Tiwari *et al.*, 2021) of 455 published models showed that about half of the models could not be reproduced using the information in the manuscripts. Without coordination, many researchers might independently try and fail to reproduce a published model, wasting a lot of time and effort. Recognising these challenges, in the emerging ModeleXchange consortium, repositories are starting to coordinate model curation and discovery. This activity should be strengthened in the context of an ELIXIR Systems Biology Community, with significant user benefits.

**Standards for design and modelling.** Less developed are the standards related to the design of experiments and the description of these designs (which are required to generate standardised, FAIR data to be subsequently capitalised on by models). This is of particular importance for complex, multistep operations that are required for many processes, such as those pertaining to biobased production of chemicals, pharmaceuticals or materials. The ESFRI programme IBISBA works on the interoperability and deployment of such concepts and standards and workflows, but many challenges remain. This is relevant for ELIXIR since these designs in the end lead to data covered in the infrastructure.

Standards for linking models with sensitive data, including electronic health records, as well as other person-related data and commercial data, with ELIXIR Core Data Resources and Deposition Databases are an identified need. ELIXIR and members of national ELIXIR Nodes are already active in defining such standards and connecting them to existing research projects in the domain as partners in the European standardisation initiative EU-STANDS4PM (European standardisation framework for data integration and data-driven in silico models for personalised medicine) that very recently has published guidelines and recommendations for data integration and model validation for computational models in the domain of clinical applications in personalised medicine ( Collin *et al.*, 2022). Through the EU-STANDS4PM initiative, ELIXIR also supports the development of a series of ISO standards with recommendations and requirements for predictive computational models in personalised medicine research ( ISO TS 9491). Such standardisation efforts need to be intensified and extended, given the increasing importance of modelling in the health domain. For this purpose, close collaborations with European initiatives, such as EU-STANDS4PM and those developing infrastructures for human digital twins (including corresponding standards for data and models) that are currently forming and will be funded in the near future, will help to jointly create a pan-European cloud-infrastructure for data integration and modelling in health research and personalised medicine.

**Interoperability between various forms of descriptive models and predictive models.** Some relevant model connections, which basically connect predictive analysis with data analysis have been developed and a few are in production. For now, most exist as proof of concept rather than production ready services. The work done in the COVID-19 Disease Map project involved much manual curation and improvement of both the converter and the source (pathway) model and can even lead to updates for the standards used. The challenge is to streamline that process and to support curators to come to more interoperable models and FAIR descriptions of provenance and evidence.

### Developing and deploying data and modelling technologies

Although the intertwining between modelling and experimentation is a hallmark of systems biology, its practical implementation remains challenging. Partly this is due to culture and insufficient training, but it also stems from difficulties in generating adequate, quantitative dynamic data that can support modelling and from the lack of models that are sufficiently accurate to handle the generated data. Addressing these challenges will require 1) an interface between big data and modelling frameworks, 2) integration of modelling approaches, including temporal and spatial modelling, and 3) application of the modelling results in a plethora of relevant domains, ranging from bioengineering at various scales to precision and personalised medicine (see Table 5).

**Table 5.** Breakdown of short, mid and long term objectives for the Technology theme.

	Aims and objectives
**Short term (~3 years)**	• How big / smart data meets models meaningfully • Intertwining temporal and spatial modelling appropriately • Interfacing to synthetic biology through model-based design and model-based-learning strategies
**Mid term (~6 years)**	• Developing good strategies (and training therein) for validating models and for checking the efficiency of tools leading to quality marks; and tools assessing predictions; • Developing theoretical and practical multi-scale modelling frameworks; • Providing the basis for developing Digital Twins (microbes, bioreactors, organs, organisms, ecosystems)
**Long term ( ~10 years)**	• Deploying Digital Twin methodologies that provide sufficiently accurate, real-time and dynamic depictions of physical biosystems • Steer and modify processes, stratify patients or support decision-making • Increase uptake of systems biology methodologies by the communities of biologists, bioengineers and physicians; • Increase the uptake of standards (e.g. for model and data reporting) by the world wide systems biology communities.

**Systems medicine.** In the systems medicine domain, additional challenges result from 1) the sensitive nature of many of the data sources, 2) the complexity of disease phenotypes and mechanisms, especially in the context of precision medicine, and 3) the ethical and legal implications of using models and model predictions in clinical decision support. Solutions will be needed that enable the use of sensitive data to build models in a manner consistent with requirements for sensitive data. The community will also facilitate the development of new models and disease maps and of improved repositories to enable their sharing in a FAIR manner, in order to address the challenge of disease complexity. Finally, the Community will engage in ELSI activities to explore the challenges of using systems medicine models close to clinical practice.

**Models as a service.** Owing to the importance of modelling resources, methods, models, data, and expertise across the board (from dynamic to constraint-based, stochastic, statistical, and data modelling) it should be an aim to enhance applications in particular by systems biology novices, many of whom are deep experts in medicine, biology or biotechnology. This should be done by providing assistance to those novices in their use of the facilities offered by the Systems Biology Community as well as by a larger number of ELIXIR Communities and as per recommendations above. It should be made easy for the novices to find the most relevant model, to adjust it to their needs, to extend it, and to even make their own new model. This relates, most immediately, to activities on Make-Me-My Model, Data for Modelling, Modelling for Data, multiscale modelling discussed above, but it will readily expand to many other ELIXIR areas.

**Digital twins of biological systems.** Systems biology models build upon data for predictions and thereby truly bring data to life. The **combination of models and smart big data** enables designing Digital Twins of biological systems and thereby strongly enhances the possibilities to explore, understand, design and predict biological behaviour. Therefore, it is proposed to provide vehicles (dedicated projects across Platforms, an ELIXIR community, or other) to smoothly integrate systems biology models with Big Data analytics and to thereby stimulate dedicated activities underpinning the development and deployment of Digital Twins for the whole range of applications in the Life Sciences. This extends from the design of highly efficient cell-based industrial and pharmaceutical processes through decision-support systems in health and disease, to integrated farming systems and ecosystem management.

### Building capacity and providing training

Whereas the separate curricula in physical sciences, data-focused life sciences, and computer science have become quite effective in training in their own disciplines, they are becoming inadequate to train multidisciplinary teams which need to tackle increasingly complex problems. Global health challenges such as non-communicable diseases, pandemics, or diseases stemming from environmental factors have all been addressed by systems biology models. When supplemented with ELIXIR earmarked information, these models should soon become ready for use in the clinic, especially because they are the only tools truly to handle the increased call from the general public for personalised medicine. This call has also been realised by the European Parliament and the European Commission. A vast increase particularly in transdisciplinary training is necessary now and ELIXIR-training is well-posed to set this up. The joint ELIXIR-ISBE course on Corona (SARS-CoV-2) epidemiology may serve as an example.

Several independent, ongoing training activities already deliver systems biology training modules within ELIXIR (e.g. Table 3). With the exponential growth of biological data, there is a lag in the identification and generation of new ones. National Training Coordinators may assist in flagging when such courses are missing from TeSS, and further promote Node-interaction when such competencies are not present by organising ELIXIR-level international courses. A possibility might be interacting with non-profit organisations working in systems biology-related areas and education such as iGEM.

Systems biologists often have to cope with scattered knowledge resources. Hence, a well-balanced and consistent set of competences are required that are compatible across the ELIXIR Nodes. We propose to implement a programme of organisational capacity building, including specific training in gap areas, advanced training, knowledge sharing and staff exchanges to build a well-developed and interconnected Systems Biology Community. We will make use of the ELIXIR’s training portal TeSS by integrating different tools and services relevant to systems biology and making it available to all Nodes. Synergising the training resources, the needs of trainees and trainers, and the communication with other ELIXIR communities is an overarching aim for the Capacity building/Training task. This will be achieved by integrating new and existing systems biology-related courses under a single umbrella towards the different objectives listed in Table 6. The corresponding activities will support the needs of current and future trainers long term, and centralise the use of systems biology materials.

**Table 6.** Breakdown of short, mid and long term objectives for Capacity building/Training theme.

	Aims and objectives
**Short term (~ 3 years)**	• Pre-screen trainees prior to training events to make recommendations for courses to be followed in the context of the event • Integrate new systems biology courses into TeSS and co-promote them with existing TeSS courses; • Strengthen synergies with the other ELIXIR Communities, e.g. via joint training events;
**Mid term (~ 6 years)**	• Extend the use of synthetic and standardised datasets in most systems biology training events • Support current and future trainers via Train the Trainer ELIXIR events
**Long term (~ 10 years)**	• Create a centralised repository of systems biology training materials aggregated by TeSS • Systematically review trends in systems biology and update the training resources accordingly

### Fostering industrial and societal embedding

Systems-level understanding and analysis of huge amounts of experimental data is required for different ‘industries’ such as hospitals, pharmaceutical industry, biotechnological companies, health care institutions, regulatory agencies, and government. However, the potential significance of systems approaches in industrial sectors has been exploited insufficiently. Systems biology aims to develop quantitative and conceptual understanding of biological phenomena. This comes with the modelling and prediction of complex processes such as functions of the human brain, ecosystem function or host-microbiome interactions. The ability to model and predict what happens to a biological system under some conditions may have a profound impact on industrial applications as diverse as the identification of the best drug candidates using PK-PD models, sustainable production of biobased chemicals and materials through model-driven designs, improvement of crop production strategies, or COVID-19 management.

Quantitative data and resources generated within IMI-funded consortia with industrial academic partnerships like TransQST, which aims to build novel systems toxicology models, have been made available through ELIXIR Core Data Resources and deposition repositories. These collaborations are valuable to ELIXIR’s Toxicology Community. Similar initiatives for systems pharmacology have been supported by IMI, major UK funding bodies and pharmaceutical companies like AstraZeneca, Pfizer and GlaxoSmithKline. Systems biology and synthetic biology will be particularly relevant to address five out of the seven key challenges identified by the European Union related to health and environmental sustainability. Hence, efforts towards transition into more environmentally sustainable economies offer unprecedented opportunities for a range of subfields of systems biology.

The ELIXIR Systems Biology Community will take an active role in the use of ELIXIR resources and in the definition of activities aiming to develop new industrial collaborations and to strengthen existing ones. These activities will be aligned to ELIXIR’s Industry Strategy by (1) facilitating collaborations between researchers in academia and industry, (2) by enabling the use of ELIXIR resources by industry and (3) by engaging effectively yet appropriately with the private sector. One way to engage with the industrial sector is through joint workshops and collaboration with other ELIXIR Communities interested in working with industry. Other objectives to achieve industrial embedding are listed in Table 7. An important aspect of these objectives is the use of Key Performance Indicators (KPIs) and "gap analysis surveys" to measure overall long-term performance and to identify priorities for improvement. The potential is enormous, given the capabilities of the deployment of data and models to describe biological systems and to enable actionable knowledge for a vast range of translational applications.

**Table 7.** Breakdown of short, mid and long term objectives for the Industrial embedding theme.

	Aims objectives
Short term (~ 3 years)	• Together with the Training Platform set up a "gap analysis survey" to find out the forces and needs for each ELIXIR Node • Implementation of turnkey solutions to different Nodes (specific training, staff exchanges, knowledge exchanges etc.) • Set up KPIs to measure the impact of different actions
Mid term (~ 6 years)	• Continue and review the process of gap analysis survey and KPIs to include SME and industry • Involve small and medium-size enterprises in the capacity building process • Identify new themes and challenges in systems biology
Long term ( ~ 10 years)	• Consolidation of the capacity building process for new partners (communities, countries etc.)

## Data Availability

No data are associated with this article.

## Footnotes

^1^ ISBE deliverables: ISBE D2.2 Preliminary Recommendations for a Data and Model Management Framework: Data and Model Stewardship for the ISBE Infrastructure (2014); ISBE D2.3 Final Recommendations for a Data and Model Management Framework: Data and Model Stewardship for the ISBE Infrastructure (2015);ISBE D2.4 Implementing Recommendations for ISBE Asset Stewardship Data and Model Stewardship for the ISBE Infrastructure (2015).

^2^ https://digital-strategy.ec.europa.eu/en/policies/1-million-genomes

^3^ See also https://omgmopodcast.com/definition-of-biotechnology/

^4^ https://www.forbes.com/sites/johncumbers/2022/09/12/white-house-inks-strategy-to-grow-trillion-dollar-us-bioeconomy

^5^ www.ibisba.eu

^6^ https://biofoundries.org; https://agilebiofoundry.org; https://www.biomade.org
