# Supplementary material for: Systems Biology in ELIXIR: modelling in the spotlight
Source: F1000Res. 2024 May 31;11:ELIXIR-1265. Originally published 2022 Nov 7. [Version 2] doi: 10.12688/f1000research.126734.2 (PMC9871403; doi:10.12688/f1000research.126734.2)
Supplement: Peer review history

# Reviewer report: Reviewer response for version 2

**DOI:** 10.5256/f1000research.165185.r285394

**Published:** 2024-07-11

**Authors:** Herbert Sauro

**Affiliations:** 1 Department of Bioengineering, University of Washington, Seattle, WA, USA

The reviewers state they have addressed my concerns However, although the authors provide the line numbers, for the changes, the manuscript has no line numbers so it was difficult to actually find the changes. After some searching I think I found the updates. One thing that I realized on a second read of the revision was that, surprisingly, there is no mention of whole-cell modeling. Although I don't believe we can model a whole cell currently (we can barely model glycolysis) a long-term effort to do so would be an enormous stimulus to the development of new math, standards, experimental techniques and modeling approaches.  I recommend the authors include this in one of the long terms goals.

Is the topic of the opinion article discussed accurately in the context of the current literature?

Yes

Are arguments sufficiently supported by evidence from the published literature?

Yes

Are all factual statements correct and adequately supported by citations?

Yes

Are the conclusions drawn balanced and justified on the basis of the presented arguments?

Yes

Reviewer Expertise:

Systems Biology

I confirm that I have read this submission and believe that I have an appropriate level of expertise to confirm that it is of an acceptable scientific standard.

# Reviewer report: Reviewer response for version 1

**DOI:** 10.5256/f1000research.139171.r156728

**Published:** 2023-01-23

**Authors:** Ioannis Androulakis

**Affiliations:** 1 Biomedical Engineering Department and Chemical & Biochemical Engineering Department, Rutgers, The State University of New Jersey, New Brunswick, NJ, USA

This is a very comprehensive report/account of systems biology activities. I do not think I have any reservations. I realize this is more of a position statement and account of capabilities and possibilities and less of a concrete discussion of specific applications. Therefore, I am assessing the manuscript as such. It does present an overwhelming picture - possibly raising concerns about the applicability - but I also realize that the purpose of this report is to present the current state of the art.

 This is admittedly a very ambitious undertaken. In that respect, I would also be curious to get the authors' opinion on whether or how such an integrative approach could eventually extend beyond large groups and integrated teams and be of assistance and value to smaller academic or research groups.

Is the topic of the opinion article discussed accurately in the context of the current literature?

Yes

Are arguments sufficiently supported by evidence from the published literature?

Yes

Are all factual statements correct and adequately supported by citations?

Yes

Are the conclusions drawn balanced and justified on the basis of the presented arguments?

Yes

Reviewer Expertise:

quantitative systems biology and pharmacology

I confirm that I have read this submission and believe that I have an appropriate level of expertise to confirm that it is of an acceptable scientific standard.

# Response

**Published:** 2024-04-17

**Authors:** John Hancock

**Affiliations:** ELIXIR, UK

We thank the reviewer for their supportive comments.

 With reference to making this initiative helpful to smaller as well as large groups, we have added two sentences on this topic (lines 326-329)

# Reviewer report: Reviewer response for version 1

**DOI:** 10.5256/f1000research.139171.r156725

**Published:** 2023-01-12

**Authors:** Herbert Sauro

**Affiliations:** 1 Department of Bioengineering, University of Washington, Seattle, WA, USA

dos Santos et al, provide a comprehensive summary of the current state of systems biology modeling as well as the challenges they see in the future within the ELIXIR framework. One of the important discussion points is suggestions for short-, mid-, and long-term goals within systems biology. The list of suggestions, however, would not have been out of place if published 20 years ago. This highlights that we still have a long way to go. For example, one of the long-term aims is improved interoperability, something that the community has been attempting to achieve for a long time. Many of the goals have a similar ring to them, though some are more interesting than others. For example, one of the mid-term goals is developing the basis for theoretical and practical multis-scale modeling. I consider this to be a major issue in multi-scale modeling which I feel urgently needs addressing. Another thoughtful goal is to use models to improve the design of experiments, that is, model-driven rather than data-driven approaches.

 Overall, the paper presents a good coverage of the efforts being undertaken in Europe and to some extent elsewhere, although some things appear to be obviously missing. For example, there is no mention of SBOL (Synthetic Biology open language) standard, even though synthetic biology is frequently mentioned in the text and is a key aspect in both the article’s short-term and mid-term goals. The biggest gap, however, is that there is very little discussion of machine learning or AI. Having said that, I should say I am firmly a card-carrying mechanistic modeler. Machine learning is a two-edged sword. On the one hand, it’s very good at finding patterns in data, but it’s also very good at hiding any understanding of why the pattern exists at all. As sentient beings, we don’t want to completely give up our intellect to a machine, however, there must be some halfway point where machine learning can inform our mechanistic models, help us determine what data we should collect (model-driven), or supply useful hypotheses to peruse. The ELIXIR program has a unique opportunity to augment its long terms goals by weaving machine learning into our mechanistic view of the world without losing our ability to understand reality. However, this is a basic research effort that will take time to develop and would probably belong to the list of long-term goals.

 Another missing component in their long-term goals is fostering tighter collaboration with the USA and other countries in the systems biology field, particularly with joint grant awards. This of course, has, and is happening. Most notably, many of the popular systems biology standards were the result of close collaboration between groups in the US and Europe. This is still very much an active area, but a statement in the list of long-term goals would help emphasize that continuing collaboration is very important to the success of the field.

 Finally, I think one of the long-term goals should be to advertise success stories more strongly. Europe, in particular, probably has more success stories in mechanistic modeling than any other trading block. This is particularly the case in understanding metabolism, where groups led by individuals such as Jacky Snoep or Bas Teusink and co-workers have made significant contributions to our understanding of energy metabolism using systems biology approaches.

 In summary, the article is interesting. It provides a status report on systems biology but perhaps misses some opportunities for future opportunities.

Is the topic of the opinion article discussed accurately in the context of the current literature?

Yes

Are arguments sufficiently supported by evidence from the published literature?

Yes

Are all factual statements correct and adequately supported by citations?

Yes

Are the conclusions drawn balanced and justified on the basis of the presented arguments?

Yes

Reviewer Expertise:

Systems Biology

I confirm that I have read this submission and believe that I have an appropriate level of expertise to confirm that it is of an acceptable scientific standard, however I have significant reservations, as outlined above.

# Response

**Published:** 2024-04-17

**Authors:** John Hancock

**Affiliations:** ELIXIR, UK

- We have added mention of SBOL, particularly where we mention the COMBINE initiative in the "Data and metadata" but also elsewhere in the document

 - With reference to AI and machine learning, We thank the reviewer for the suggestion, which has been added into the manuscript (lines 221-222)

 - With reference to fostering closer links to the US, this has also been added as a long-term goal (lines 223-224).

 - With reference to advertising success stories, we added this as a mid-term goal (line 203)

# Reviewer report: Reviewer response for version 1

**DOI:** 10.5256/f1000research.139171.r155195

**Published:** 2022-11-28

**Authors:** Yoram Vodovotz

**Affiliations:** 1 Department of Surgery, University of Pittsburgh, Pittsburgh, PA, USA

Dos Santos et al present a white paper outlining the overall rationale, current work, and future plans of the ELIXIR consortium.  The manuscript is overall logical and well-written. However, there are some points that the authors should consider and that would improve the manuscript overall:

1. Overall, the thrust of the manuscript and ELIXIR as a whole seems to center on the concept that Systems Biology is predicated on the generation of mechanistic mathematical models, with datasets envisioned as being used for calibration/verification/validation of these mechanistic models. While this is certainly a major goal for many in this field, there seems to be little mention of machine learning (i.e. data-driven modeling) as an alternative (or, ideally, an adjunct approach that could synergize with or integrate machine learning with mechanistic modeling), though there is mention of a focus group on machine learning and a short term goal stated as “Understanding how big/smart data meets models meaningfully.” It would seem that there is a need to establish workflows and pipelines wherein data are generated, data-driven modeling/machine learning is performed to identify important features in the datasets, and those features are, in some way, included in the resultant mechanistic models. In this way, the current gap between data-driven and mechanistic modeling could be bridged. This is alluded to, but not made explicit, in the section entitled “Using descriptive models and linking them to predictive models” and is a component of Figure 3. While I realize that this white paper encapsulates and presents the current consensus within the ELIXIR community, a more explicit, high-level overview of how data-driven and mechanistic modeling could interface (e.g. as noted in Table 5 under “short-term goals”) would improve the manuscript. Perhaps the authors need to clarify exactly what the term “smart big data” means, because it is possible that this term refers to what I have mentioned above.
2. Executive Summary, definition of Systems Biology: “thousands” should probably be changed to “myriad” or some other more general term since biological interactions could add up to the millions or more. For example, while there are thousands of genes, there are millions of single-nucleotide polymorphisms that could impact biological systems.
3. Executive Summary: I think the authors presuppose that readers are already familiar with ELIXIR and its myriad activities. However, many readers will not be aware of this group. Thus, there should be a straightforward introduction to ELIXIR and its core mission. In a related issue, the authors should make sure to state that their focus is predominantly on developments happening in Europe since that is where this consortium is located, though the white paper does cite resources that were developed in the U.S.
4. Executive Summary, Tables1/2: The authors are to be lauded for their extensive listing of prior/existing Systems Biology initiatives. However, Avicenna Alliance is not mentioned (though it is closely related to VPH), and this group has made major strides in driving the adoption of computational modeling and in silico clinical trials.
5. Introduction: in the section entitled “Systems biology underpinning systems medicine,” the reader may be well-served by mentioning Translational Systems Biology and relevant publications from that related field. This could be included also in the section on Quantitative and Systems Pharmacology. A discussion of both of these approaches is of relevance to the white paper’s focus on in silico clinical trials and digital twins. In this regard, the lack of connection between the Systems Medicine and QSP sections is a bit of missed opportunity since at the end of the day drugs are being developed to treat patients, and the disease models that serve as the basis of digital twins are offshoots of the same models used to develop the drugs in the first place (e.g. the goals and objectives listed under Table 4).
6. Minor:
  1. Introduction: “…the study objects of systems biology”; I assume the authors mean “objectives”?

Is the topic of the opinion article discussed accurately in the context of the current literature?

Partly

Are arguments sufficiently supported by evidence from the published literature?

Yes

Are all factual statements correct and adequately supported by citations?

Yes

Are the conclusions drawn balanced and justified on the basis of the presented arguments?

Partly

Reviewer Expertise:

Systems biology; inflammation; computational biology; immunology

I confirm that I have read this submission and believe that I have an appropriate level of expertise to confirm that it is of an acceptable scientific standard, however I have significant reservations, as outlined above.

# Response

**Published:** 2024-04-17

**Authors:** John Hancock

**Affiliations:** ELIXIR, UK

1. We thank the reviewer for summarising the perspective on mechanistic models and AI. Mechanistic models are indeed a major goal for the field. For many years, perhaps decades, they have provided a clear-box approach to modelling, allowing the researchers to be in control of the abstractions, and facilitating a transparent mapping to biological processes. AI models, on the other hand, are prone to create difficulties in understanding their inner workings, and much more so in the large language models developed in recent years. As suggested, interfacing the two approaches could be a way to bridge the gap between the explainable and unexplainable modelling approaches. As an infrastructure Community, we will closely follow the latest research developments in this regard. The manuscript has been adjusted by replacing the term “big/smart data” with “big data and AI” (lines 171 and 575) and to include the above expansion on the interfacing between modelling approaches (lines 1220-1229).

 2. The suggestion has been applied (line 50).

 3. We have added a brief description of ELIXIR as a European infrastructure to this section (lines 63-66)

 4. Avicenna Alliance has been added to table 2 (line 1546).

 5. We have tried to address this point in the text, especially in the section discussing Industrial Embedding and PBPK models

 6. The authors did indeed intend to discuss “study objects”, that is the objects studied by systems biology (line 244)

# Response

**Published:** 2024-06-20

**Authors:** Yoram Vodovotz

**Affiliations:** University of Pittsburgh, USA

I thank the authors for addressing my core concerns.
